# H5N1 virus invades the mammary glands of dairy cattle through ‘mouth-to-teat’ transmission

**DOI:** 10.1093/nsr/nwaf262

**Published:** 2025-07-01

**Authors:** Jianzhong Shi, Huihui Kong, Pengfei Cui, Guohua Deng, Xianying Zeng, Yongping Jiang, Xijun He, Xianfeng Zhang, Lei Chen, Yichao Zhuang, Yan Wang, Jinming Ma, Jiaqi Li, Yaping Zhang, Congcong Wang, Chen He, Jiaxin Yang, Jiongjie Li, Wenyu Liu, Jinyu Yang, Shenggang Mao, Jinxiong Liu, Pucheng Chen, Guobin Tian, Chengjun Li, Yuntao Guan, Zhigao Bu, Hualan Chen

**Affiliations:** State Key Laboratory of Animal Disease Control, Harbin Veterinary Research Institute, Chinese Academy of Agricultural Sciences, Harbin 150069, China; State Key Laboratory of Animal Disease Control, Harbin Veterinary Research Institute, Chinese Academy of Agricultural Sciences, Harbin 150069, China; State Key Laboratory of Animal Disease Control, Harbin Veterinary Research Institute, Chinese Academy of Agricultural Sciences, Harbin 150069, China; State Key Laboratory of Animal Disease Control, Harbin Veterinary Research Institute, Chinese Academy of Agricultural Sciences, Harbin 150069, China; State Key Laboratory of Animal Disease Control, Harbin Veterinary Research Institute, Chinese Academy of Agricultural Sciences, Harbin 150069, China; State Key Laboratory of Animal Disease Control, Harbin Veterinary Research Institute, Chinese Academy of Agricultural Sciences, Harbin 150069, China; National High Containment Laboratory for Animal Disease Control and Prevention, Harbin 150069, China; National High Containment Laboratory for Animal Disease Control and Prevention, Harbin 150069, China; State Key Laboratory of Animal Disease Control, Harbin Veterinary Research Institute, Chinese Academy of Agricultural Sciences, Harbin 150069, China; State Key Laboratory of Animal Disease Control, Harbin Veterinary Research Institute, Chinese Academy of Agricultural Sciences, Harbin 150069, China; State Key Laboratory of Animal Disease Control, Harbin Veterinary Research Institute, Chinese Academy of Agricultural Sciences, Harbin 150069, China; State Key Laboratory of Animal Disease Control, Harbin Veterinary Research Institute, Chinese Academy of Agricultural Sciences, Harbin 150069, China; State Key Laboratory of Animal Disease Control, Harbin Veterinary Research Institute, Chinese Academy of Agricultural Sciences, Harbin 150069, China; State Key Laboratory of Animal Disease Control, Harbin Veterinary Research Institute, Chinese Academy of Agricultural Sciences, Harbin 150069, China; State Key Laboratory of Animal Disease Control, Harbin Veterinary Research Institute, Chinese Academy of Agricultural Sciences, Harbin 150069, China; State Key Laboratory of Animal Disease Control, Harbin Veterinary Research Institute, Chinese Academy of Agricultural Sciences, Harbin 150069, China; State Key Laboratory of Animal Disease Control, Harbin Veterinary Research Institute, Chinese Academy of Agricultural Sciences, Harbin 150069, China; State Key Laboratory of Animal Disease Control, Harbin Veterinary Research Institute, Chinese Academy of Agricultural Sciences, Harbin 150069, China; State Key Laboratory of Animal Disease Control, Harbin Veterinary Research Institute, Chinese Academy of Agricultural Sciences, Harbin 150069, China; Harbin Weike Biotechnology Co., Ltd., Harbin 150069, China; Harbin Weike Biotechnology Co., Ltd., Harbin 150069, China; State Key Laboratory of Animal Disease Control, Harbin Veterinary Research Institute, Chinese Academy of Agricultural Sciences, Harbin 150069, China; State Key Laboratory of Animal Disease Control, Harbin Veterinary Research Institute, Chinese Academy of Agricultural Sciences, Harbin 150069, China; State Key Laboratory of Animal Disease Control, Harbin Veterinary Research Institute, Chinese Academy of Agricultural Sciences, Harbin 150069, China; State Key Laboratory of Animal Disease Control, Harbin Veterinary Research Institute, Chinese Academy of Agricultural Sciences, Harbin 150069, China; National High Containment Laboratory for Animal Disease Control and Prevention, Harbin 150069, China; State Key Laboratory of Animal Disease Control, Harbin Veterinary Research Institute, Chinese Academy of Agricultural Sciences, Harbin 150069, China; National High Containment Laboratory for Animal Disease Control and Prevention, Harbin 150069, China; State Key Laboratory of Animal Disease Control, Harbin Veterinary Research Institute, Chinese Academy of Agricultural Sciences, Harbin 150069, China

**Keywords:** H5N1 avian influenza virus, dairy cattle, replication, transmission, vaccination

## Abstract

H5N1 influenza outbreaks have been reported on more than 1070 dairy farms across 17 states in the USA. Damage to the mammary gland and high levels of virus in milk were common features of the infected cattle, but it is unclear how the virus initially invades the mammary glands, and no control strategy is currently available. Here, we found that cattle oral tissues support H5N1 virus binding and replication, and virus replicating in the mouth of cattle transmitted to the mammary glands of dairy cattle during sucking. We also found that an H5 inactivated vaccine or a hemagglutinin-based DNA vaccine induced sterilizing immunity in cows against challenges with different H5N1 viruses. Our study provides insights into H5N1 virus transmission and control in cattle.

## INTRODUCTION

On 25 March 2024, H5N1 virus infection was reported in dairy cattle in Texas, USA. Studies indicated that the outbreak was caused by a clade 2.3.4.4b H5N1 virus of the B3.13 genotype, a reassortant of an ancestral European 2.3.4.4b virus and North American wild bird avian influenza viruses [1–4]. Respiratory symptoms of infected cattle were limited, but reduced milk production and mastitis with high levels of virus in mammary glands and milk were common features of the infected cattle, and up to 10% mortality was recorded in dairy herds [5–8]. Despite major efforts to control the outbreak, H5N1 virus infection in dairy cattle continues to spread. As of 7 June 2025, H5N1 virus infection has been reported in over 1070 dairy farms across 17 states [9], with cow-to-cow transmission speculated to be mediated by the milking process [10–12]. Cattle H5N1 viruses have spilled over into domestic felines, alpacas, poultry and house mice [6,13–15], and also into several different wild mammals [16,17]. Of the 70 human infection cases reported in USA in 2024, 41 involved dairy workers [9], raising global public health concerns. In this study, we asked two important questions: (i) how does the H5N1 virus get to the mammary glands; and (ii) could vaccination prevent H5N1 virus infection and spread in cattle?

## RESULTS

### Replication of Eurasia H5N1 avian viruses in dairy cattle after intranasal inoculation

All experiments with H5N1 influenza viruses were performed in the Animal Biosafety Level 3+ (ABSL-3+) facility in Harbin Veterinary Research Institute, China. To investigate tissue distribution, especially whether H5N1 viruses can invade the mammary glands of cattle after intranasal infection, we first evaluated the replication in cows of two H5N1 viruses detected in China recently: A/tundra swan/Fujian/SD121/2023 (TS/23 virus) and A/duck/Guizhou/S1711/2024 (DK/24 virus). Both viruses bear the HA gene of clade 2.3.4.4b, and their genome similarity and phylogenetic relationship with the index cattle virus reported in the USA are shown in Table S1 and Fig. S1. Two groups of three lactating dairy cattle obtained from a local farm were respectively intranasally inoculated with 2 mL of 10^6^ 50% egg infectious dose (EID_50_)/mL of virus. One animal from each group was euthanized on Days 3, 6 and 14 post-inoculation (p.i.), respectively, and their organs and tissues, including nasal turbinate, soft palate, root of tongue, larynx, tonsils, sublingual gland, parotid gland, submandibular gland, submandibular lymph nodes, trachea, bronchus, lungs, mammary glands, heart, liver, spleen, pancreas, kidneys, brain, uterus, ovaries, bladder, thymus gland, blood and different parts of the digestive tract, were collected to test for virus replication. Swabs (nasal, oral and rectal) and milk from different mammary glands of each live animal were collected every day from Day 1 p.i. to monitor virus shedding.

**Figure 1.**
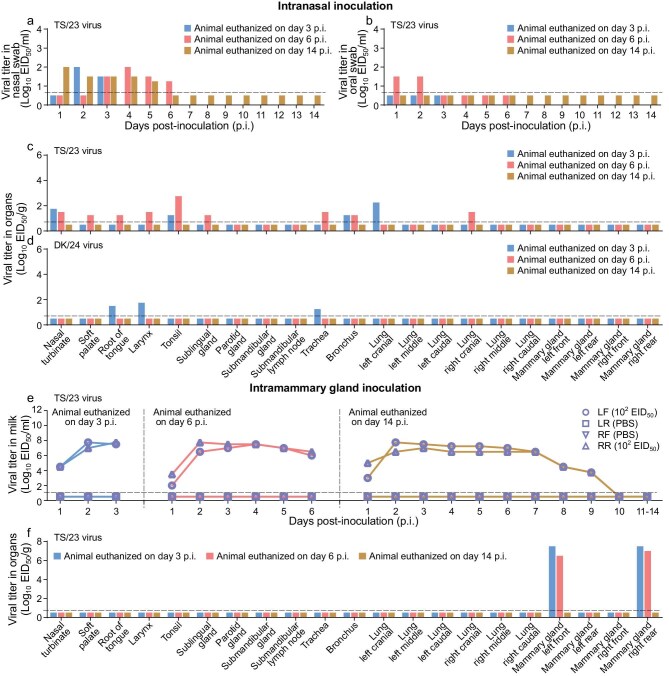
Replication of Eurasia avian H5N1 viruses in dairy cows. Cows were intranasally inoculated with the TS/23 virus (a–c) or DK/24 virus (d), and one group of cattle was also infected via intramammary gland inoculation with the TS/23 virus (e, f). Virus in the swabs, milk and tissues of cows was titrated in eggs. Viral titers in the nasal swabs (a), oral swabs (b) and tissues (c) of cows infected intranasally with the TS/23 virus are shown. (d) Viral titers in tissues of cows infected intranasally with DK/24 virus. (e) Viral titers in milk from different udders of cattle infected through the intramammary glands. (f) Viral titers in tissues of cattle infected through the intramammary glands. Virus-negative swabs and tissues are shown in Figs S2 and S4. Each color bar represents the value of an individual animal. The horizontal dashed lines in the panels indicate the lower limit of detection. PBS, phosphate buffered saline.

Cattle showed no body temperature increase or obvious symptoms during the observation period (Table S2). In the TS/23 virus-inoculated group, virus shedding was detected in the nasal swabs of all three animals (Fig. 1a) and in the oral swabs of one animal (Fig. 1b) but was not detected from any rectal swabs or milk (Fig. S2a and e). In the animal euthanized on Day 3 p.i., virus was detected in the nasal turbinate, tonsil, bronchus and one lung lobe, but was not detected in any other organs or tissues. In the animal euthanized on Day 6 p.i., virus was detected in the nasal turbinate, soft palate, root of tongue, tonsil, larynx, sublingual gland, trachea, bronchus and one lung lobe, but was not detected in any other organs or tissues. In the animal euthanized on Day 14, virus was not detected in any organs or tissues tested (Fig. 1c and Fig. S2g). In the DK/24 virus-inoculated group, virus was detected in the root of tongue, larynx and trachea of the animal that was euthanized on Day 3 p.i., but was not detected in any swabs or any other organs or tissues collected from the animals that were euthanized on Days 6 and 14 p.i. (Fig. 1d and Fig. S2b, c, d, f and h) These findings indicate that the DK/24 virus has very limited replicative ability in cattle, even when inoculated intranasally at a high dose. TS/23 and DK/24 differ by 105 amino acids in their 11 proteins (Fig. S3), and it is unclear which ones are potential molecular markers that differentiate the viral replicative efficiency of these two viruses in dairy cows.

### Replication of TS/23 virus in dairy cattle after intramammary gland inoculation

Virus replication in the mammary gland is a harmful feature of the US cattle H5N1 virus. To investigate whether the mammary gland of cattle is susceptible to H5N1 virus, three lactating cattle were each inoculated with a low dose of the TS/23 virus: 1 mL (10^2^ EID_50_/mL) to the left front mammary gland and 1 mL to the right rear mammary gland. Animal euthanasia timepoints and sample collection were the same as those used for the intranasally inoculated animals described above. Two cattle showed a slight increase in body temperature and the milk from their virus-inoculated udders became yellow and thick (Table S2); no other symptoms were observed in these animals. We did not find virus in any swabs of any cattle (Fig. S4a–c), but detected virus in milk collected from the two inoculated mammary glands (left front and right rear) of all three animals as early as Day 1 p.i. Virus shedding in milk lasted for up to 9 days p.i. in the animal that was euthanized on Day 14 p.i. (Fig. 1e). Virus was detected in the inoculated mammary glands of the animals that were euthanized on Days 3 and 6 p.i., with titers of up to 10^7.5^ EID_50_/g, but was not detected in the mammary glands of the animal that was euthanized on Day 14 p.i., or in any other tissues collected from the animals (Fig. 1f and Fig. S4d), indicating that the mammary gland of cattle is susceptible to TS/23 virus, but virus in inoculated mammary gland cannot migrate to neighboring mammary glands.

### Replication of North America H5N1 cow virus in dairy cattle after intranasal infection

**Figure 2.**
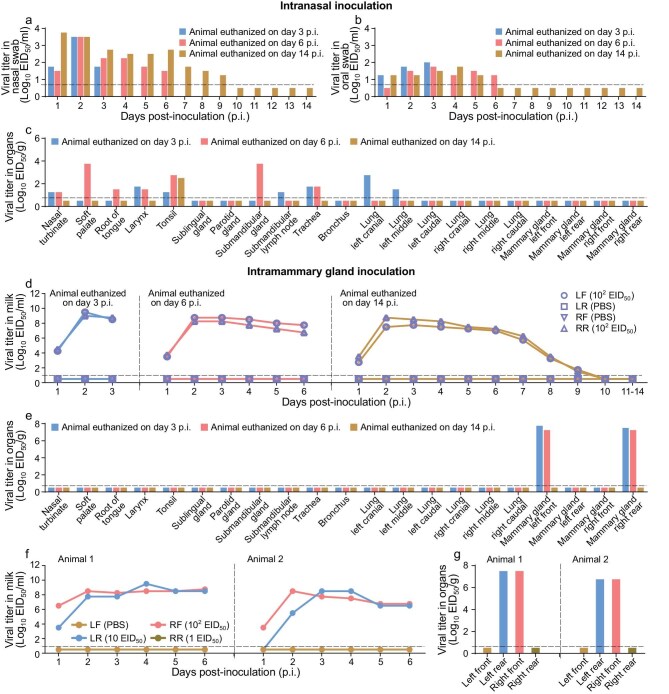
Replication of DC/24 virus in dairy cows. Cows were inoculated with 2 × 10^6^ EID_50_ of DC/24 virus intranasally (a–c), with 2 × 10^2^ EID_50_ of DC/24 via the intramammary glands (d, e), or with the indicated different doses of DC/24 virus to the indicated different mammary glands (f, g), and virus in the swabs, milk and tissues of cows was titrated in eggs. Viral titers in the nasal swabs (a), oral swabs (b) and tissues (c) of cows infected intranasally. (d, f) Viral titers in milk from different udders of cattle infected through the intramammary glands. (e, g) Viral titers in tissues of cattle infected through the intramammary glands. Virus-negative swabs and tissues are shown in Fig. S5. Each color bar represents the value of an individual animal. The horizontal dashed lines in the panels indicate the lower limit of detection. PBS, phosphate buffered saline.

The above studies indicated that even with high doses of intranasal inoculation, the H5N1 avian influenza viruses could not reach the mammary glands. Cattle H5N1 virus has been detected in the mammary glands of ferrets following intranasal inoculation [18,19]; however, it remains unclear whether the cattle H5N1 virus could reach the mammary glands in lactating cattle following intranasal infection. To answer this question, we generated the index cattle H5N1 virus, A/dairy cow/Texas/24-008749-001/2024 (DC/24), by reverse genetics and assessed it in lactating dairy cattle. Three cattle were each intranasally inoculated with 2 mL of the virus (10^6^ EID_50_/mL). Animal treatment and sample collection were the same as those for the TS/23 virus-inoculated animals. One cow had a fever on Day 4 p.i. (Table S2). Virus was detected in nasal swabs collected at all timepoints from the cattle that were euthanized on Days 3 and 6 p.i., and in nasal swabs collected between Days 1 and 9 p.i. of the animal that was euthanized on Day 14 p.i. (Fig. 2a). Virus was also detected in oral swabs from three animals on several occasions between Days 1 and 6 p.i. (Fig. 2b), but was not detected in rectal swabs or milk (Fig. S5a and b). In the animal euthanized on Day 3 p.i., virus was detected in the nasal turbinate, larynx, tonsil, submandibular lymph nodes, trachea and two different lung lobes, but was not detected in any other organs or tissues. In the animal euthanized on Day 6 p.i., virus was detected in the nasal turbinate, soft palate, root of tongue, tonsil, larynx, submandibular gland and trachea, but was not detected in any other organs or tissues. In the animal euthanized on Day 14, virus was detected in the tonsil but not in any other organs or tissues tested (Fig. 2c and Fig. S5c).

### Replication of DC/24 virus in dairy cattle after intramammary gland inoculation

The above study indicated that the DC/24 virus cannot reach cattle mammary gland following intranasal infection. We therefore performed the intramammary gland-inoculated study as we did for the TS/23 virus. Two cattle had increased body temperature on Days 2 and 3 p.i., and the milk from their virus-inoculated udders became yellow and thick (Table S2). Virus was detected in the milk collected from the infected mammary glands (left front and right rear) of all three animals, and virus shedding in milk lasted for 9 days in the animal that was euthanized on Day 14 p.i., with peak titers about 10-fold higher than that of the TS/23 virus-inoculated cattle (Fig. 2d versus Fig. 1e). Virus was detected in the left front and right rear mammary glands of the animals that were euthanized on Days 3 and 6 p.i. (Fig. 2e), but not in any other samples collected from these two animals (Fig. S5d–f), or any swabs or organs from the animal that was euthanized on Day 14 p.i. (Fig. 2e and Fig. S5g).

We further investigated the minimum dose required for the DC/24 virus to infect the mammary glands. Two cattle were respectively inoculated with three different doses of the virus into three different mammary glands: 1 mL of 10^2^ EID_50_ to the right front teat, 1 mL of 10 EID_50_ to the left rear teat, and 1 mL of 1 EID_50_ to the right rear teat. Virus was detected in the milk and tissues of the right front and left rear udders but was not detected in the milk or tissues collected from the left front and right rear udders (Fig. 2f and g), indicating that 10 EID_50_ of the DC/24 virus is required for successful infection of the mammary gland of cattle.

These studies demonstrate that replication of H5N1 influenza viruses in cows varies among strains. Virus infected intranasally can only replicate in tissues of the mouth and respiratory tract of the cattle, and virus inoculated into the mammary gland replicates only in the inoculated gland but does not migrate to neighboring mammary glands of the cattle. Therefore, entry through the teat is the only natural way the virus can infect the mammary gland of cattle.

### Sialic acid receptor distribution in different cattle tissues

**Figure 3.**
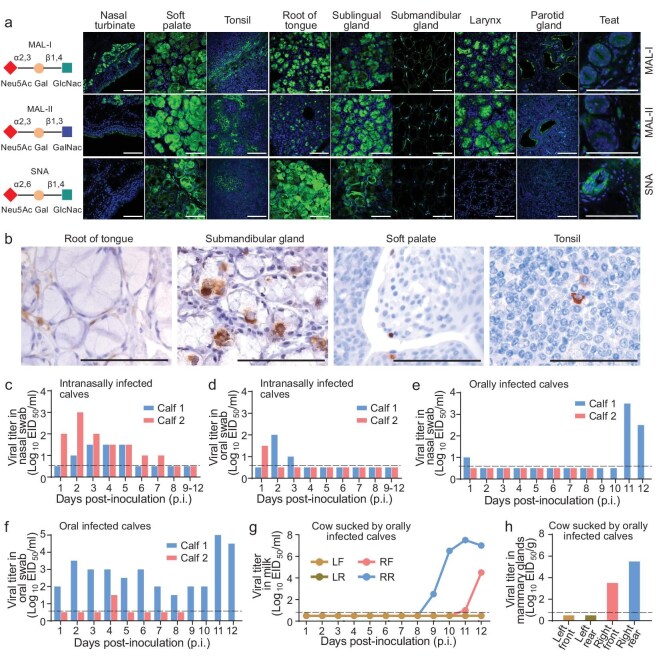
‘Mouth-to-teat’ transmission of H5N1 virus in cattle. (a) Receptor distribution in different tissues of cattle. Tissues of cattle were stained with three fluorescein-labeled lectins: MAL-I and MAL-II stain different avian-type receptors, and SNA stains human-type receptors. (b) Viral antigen in oral tissues collected from cattle intranasally infected with DC/24 and euthanized on Day 6 p.i. was analyzed by immunohistochemistry. Scale bars = 100 µm. (c–h) Viral titers in the swabs of calves and in milk and tissues of cows. Viral titers in nasal swabs (c) and oral swabs (d) of intranasally infected calves. Viral titers in nasal swabs (e) and oral swabs (f) of orally infected calves. (g) Viral titers in milk of cows sucked by orally infected calves. (h) Viral titers in mammary glands of cows sucked by orally infected calves. Each color bar represents the value of an individual animal. The horizontal dashed lines in the panels indicate the lower limit of detection.

Our studies indicated that the H5N1 virus could replicate in multiple oral tissues, lungs and mammary glands. Binding to sialic acid receptors on the cell surface is the first step for influenza virus to infect cells, and there are mainly two types of sialic acid receptors: α2,6-linked sialic acids (also known as the human-type receptor) and α2,3-linked sialic acids (also known as the avian-type receptor). We therefore investigated the types of sialic acid receptors in different tissues of cattle. We found that cells in the soft palate, tonsils, root of tongue, sublingual glands, submandibular glands, lungs, teats and mammary glands of cattle express both avian-type sialic acid receptors [Maackia amurensis lectin I (MAL-I) or MAL-II stain-positive] and human-type [Sambucus nigra lectin (SNA) stain-positive] sialic acid receptors, whereas the nasal turbinate, larynx, trachea and parotid glands of cattle only express high levels of avian-type receptors (Fig. 3a and Fig. S6). This receptor distribution allows viruses that can bind to either avian-type or human-type receptors to attach to the cells of oral tissues.

The DC/24 virus was detectable in oral swabs of dairy cattle for up to 6 days with a titer of approximately 10^2^ EID_50_ (Fig. 2b). Replication of the virus in oral tissues was further confirmed by immunohistochemistry studies on the virus-positive oral tissues of the DC/24 virus-infected cattle, and the amount of antigen in the root of tongue and submandibular gland was notably higher than that in the tonsil and soft palate (Fig. 3b). The high level of sialic acid receptors and the efficient replication of the virus in certain oral tissues of cattle not only supports influenza virus infection through contaminated feed or water, but also makes the oral cavity an ideal site for H5N1 virus to evolve and transmit.

### ‘Mouth-to-teat’ transmission of H5N1 virus in cattle

Since the DC/24 virus replicated efficiently in certain tissues of cattle mouth, and given that some lactating cattle ‘steal milk’ through self-nursing or mutual nursing [20–26] (Fig. S7), we speculated that ‘mouth-to-teat’ transmission may be the route by which the H5N1 virus initially infects the mammary glands of dairy cows. To test this hypothesis, we infected a pair of calves with 10^6^ EID_50_ of the DC/24 virus intranasally and another pair orally. Six hours later, each pair of calves was housed with a lactating cow in one room (Fig. S8a), and allowed to suck the teats as frequently as needed. Nasal and oral swabs from the calves and the milk from the lactating cow were collected every day for 12 days for virus titration in eggs. Body temperature and milk color were monitored daily (Table S3).

In one of the intranasally inoculated calves, virus was detected in nasal swabs from Days 1 to 7 p.i. and in the oral swab on Day 1 p.i.; in the other intranasally inoculated calf, virus was detected in nasal swabs from Days 2 to 5 p.i. and in oral swabs on Days 2 and 3 p.i. (Fig. 3c and d), but virus was not detected in the milk or mammary gland tissues collected from the lactating cow that was housed with these two calves (Fig. S8b and c). In one of the orally inoculated calves, virus was only detected in the oral swabs on Day 4 p.i. in one calf, which we euthanized on Day 8 p.i. to give the other calf more opportunities to suck. In the other orally inoculated calf, virus was detected in the nasal swabs on Day 1 p.i. and Days 11 and 12 p.i., and in the oral swabs from Day 1 to Day 12 p.i. (Fig. 3e and f). In the lactating cow that was housed with the orally infected calves, virus was detected in the milk from one udder on Days 9 to 12 after the calves were infected and another udder on Days 11 and 12 after the calves were infected (Fig. 3g). Virus was also detected in the tissues collected from these two udders of the cow that was euthanized at the end of the study (Fig. 3h). Of note, the increase in the virus titer in the oral swabs on Days 11 and 12 p.i., as well as the recurrence of the virus in the nasal swabs on Days 11 and 12 p.i., of calf 2 (Fig. 3e and f) may be related to the consumption of milk containing the virus. Our results show that H5N1 virus in the oral cavity of cattle can spread to the mammary glands of cattle during nursing.

### Antibody response induced by inactivated and DNA vaccines

Vaccination has been a strategy for control of highly pathogenic avian influenza in China since 2004, and studies indicate that antigenically well-matched vaccine can provide complete protection in poultry against highly pathogenic avian influenza virus challenge [27–33]. Could vaccination also prevent H5N1 influenza infection in cattle? To answer this question, we tested two vaccines (an inactivated vaccine and an HA-based DNA vaccine) in dairy cattle. The HA gene of both vaccines was derived from a clade 2.3.4.4b virus we detected in a swan in China in 2020 [34,35]. Groups of six lactating cattle obtained from local farms were inoculated with two doses of inactivated vaccine or DNA vaccine in 4 mL, with a 3-week interval between vaccinations. Sera and milk were collected weekly for antibody detection. The vaccines did not cause any side effects in the cattle.

In the H5 inactivated vaccine-inoculated cattle, hemagglutination inhibition (HI) antibody was detected in sera, and neutralization (NT) antibody was detected in sera and milk as little as 2 weeks after the first inoculation dose (Table S4), with titers comparable with that in cattle that received high-dose live virus inoculation (Table S5). The antibody response was slower in some of the DNA vaccine-inoculated cattle, and the titers were also relatively lower than those in the inactivated vaccine-inoculated cattle (Table S4). After the second dose, there was a clear increase in antibody levels (Table S4). The antibody titers against the TS/23 and DC/24 viruses were comparable to that against the vaccine strain (Table S6). Further analysis showed that the antibodies in sera and milk induced by the two vaccines were mainly IgG (Fig. S9).

### Protective efficacy of vaccines in cattle against TS/23 virus challenge

Two weeks after the second dose, three cattle from each vaccinated group along with three unvaccinated control cattle were transferred to the ABSL-3+ facility and challenged with the TS/23 virus. To investigate whether vaccination could prevent the cattle from high-dose intranasal exposure and possible ‘mouth-to-teat’ infection, the cattle were simultaneously challenged with 2 mL of virus via the intranasal route (10^6^ EID_50_/mL, 1 mL/nostril) and 2 mL of virus via the intramammary gland route (10^2^ EID_50_/mL, 1 mL per teat). One animal from each group was euthanized on Days 3, 6 and 12 post-challenge (p.c.), respectively.

**Figure 4.**
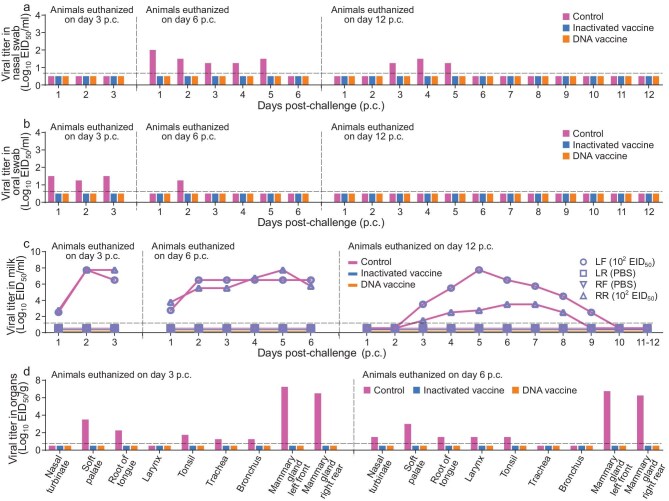
Protective efficacy of different vaccines in cattle against TS/23 virus challenge. Vaccinated cattle and control cattle were challenged with TS/23 virus through both intranasal and intramammary gland inoculation, and samples, including nasal swabs, oral swabs, milk and tissues were collected for virus titration in eggs. (a) Viral titers in nasal swabs. (b) Viral titers in oral swabs. (c) Viral titers in milk. (d) Viral titers in tissues of cattle euthanized on Days 3 and 6 p.c. Virus-negative tissues are shown in Fig. S10. Each color bar represents the value of an individual animal. The horizontal dashed lines in the panels indicate the lower limit of detection. PBS, phosphate buffered saline.

In the control group, two cattle had body temperature increase on Days 2 and 3 p.i., respectively, and milk from their virus-inoculated udders started to turn yellow and thick on Day 3 p.i. (Table S7). Virus was detected in the nasal swabs collected from the two cattle that were euthanized on Days 6 and 12 p.c. (Fig. 4a), in the oral swabs collected from the two cattle that were euthanized on Days 3 and 6 p.c. (Fig. 4b) and in the milk collected from the inoculated mammary glands of all three cattle (Fig. 4c). Virus was also detected in some tissues of the mouth and respiratory tract, and the virus-inoculated mammary glands of the two cattle that were euthanized on Days 3 and 6 p.c., but not in any tissues collected from the cow that was euthanized on Day 12 p.c. (Fig. 4d and Fig. S10). In contrast, virus was not detected in any samples collected from the animals in the vaccinated groups (Fig. 4a–d and Fig. S10). Cattle did not have fever, and their milk remained normal (Table S7). Histological damage or virus antigen was not detected in the lungs or mammary glands of the vaccinated animals (Supplementary text, Fig. S11).

### Protective efficacy of vaccines in cattle against DC/24 virus challenge

**Figure 5.**
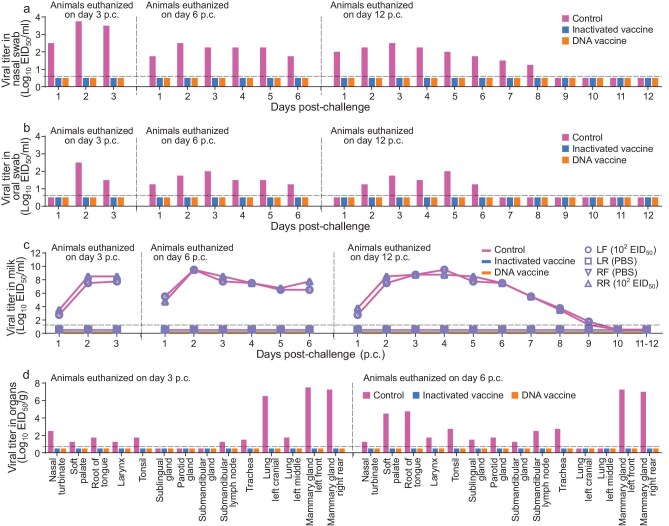
Protective efficacy of different vaccines in cattle against DC/24 virus challenge. Vaccinated cattle and control cattle were challenged with DC/24 virus through both intranasal and intramammary gland inoculation, and samples, including nasal swabs, oral swabs, milk and tissues, were collected for virus titration in eggs. (a) Viral titers in nasal swabs. (b) Viral titers in oral swabs. (c) Viral titers in milk. (d) Viral titer in tissues of cattle euthanized on Days 3 and 6 p.c. Virus-negative tissues are shown in Fig. S12. Each color bar represents the value of an individual animal. The horizontal dashed lines in the panels indicate the lower limit of detection.

To investigate whether the vaccines could also prevent infection against the cattle-adapted H5N1 virus, we performed a similar challenge study with the DC/24 virus. The animals received two doses of vaccine and the challenge was performed at 5 weeks after the second dose, which occurred 3 weeks after the TS/23 virus challenge due to the limitations of laboratory space. Serum and milk antibody titers of the cattle are shown in Table S4. In the control group, two cattle had body temperature increase, and milk from their virus-inoculated udders started to turn yellow and thick on Days 3 or 4 p.i. (Table S7). Virus was detected in the nasal swabs (Fig. 5a), oral swabs (Fig. 5b) and milk collected from the inoculated mammary glands of all three cattle (Fig. 5c). Virus was also detected in some tissues of the mouth and respiratory tract, and the two virus-inoculated mammary glands of the two cattle that were euthanized on Days 3 and 6 p.c., but not in other tissues of these two animals (Fig. S12a and b) or any tissues collected from the cattle that was euthanized on Day 12 p.c. (Fig. 5d and Fig. S12c). In contrast, virus was not detected in any swabs or tissues collected from the vaccinated animals (Fig. 5a–d and Fig. S12a and b), and the cattle did not have fever or changes in milk color or thickness (Table S7).

### Protective efficacy of vaccines in cattle against high-dose H5N1 virus challenge via intramammary gland inoculation

In the above challenge studies, we used a low dose for the intramammary inoculation to investigate whether the vaccines could prevent the possible ‘mouth-to-teat’ transmission of H5N1 virus in cattle. Once virus has entered the mammary glands, the milking process likely spreads the virus within the herd [10,36,37]. Virus titers in the milking equipment may be higher than those in the oral cavity of cattle, so could vaccination also prevent virus transmission during the milking process? To answer this question, groups of three vaccinated and control cattle were challenged via intramammary gland inoculation with three different doses of the DC/24 virus to three different mammary glands: 1 mL of 10^2^ EID_50_ to the left rear teat, 1 mL of 10^4^ EID_50_ to the right front teat and 1 mL of 10^6^ EID_50_ to the right rear teat.

**Figure 6.**
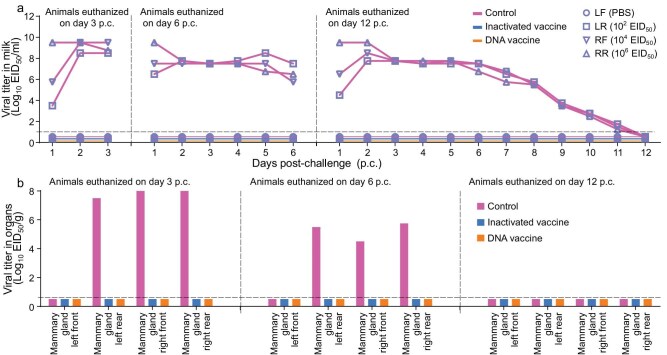
Protective efficacy of different vaccines in cattle against high-dose DC/24 virus intramammary gland challenge. Vaccinated cattle and control cattle were challenged with DC/24 virus through intramammary gland inoculation with the indicated doses, and milk and tissues of mammary glands were collected for virus titration in eggs. (a) Viral titers in milk. (b) Viral titers in tissues of mammary glands of cattle euthanized on different days p.c. Each color bar represents the value of an individual animal. The horizontal dashed lines in the panels indicate the lower limit of detection.

In the control group, body temperature increase was detected in two animals and milk changes were observed in all three animals (Table S7). Virus was detected in the milk collected from the three virus-inoculated udders of all three animals (Fig. 6a) and in tissues from the virus-inoculated mammary glands of the cattle that were euthanized on Days 3 and 6 p.c., but not in the tissues collected from the animal that was euthanized on Day 12 p.c. (Fig. 6a and b). Importantly, in both vaccinated groups, the animals did not have fever after the virus challenge (Table S7), and virus was not detected in any milk samples or tissues collected from these animals (Fig. 6a and b), indicating that the vaccinated cattle were completely protected against high-dose intramammary gland challenge with H5N1 virus.

## DISCUSSION

Although previous studies have detected cattle H5N1 virus in the mammary glands of ferrets following intranasal inoculation [18,19], in the present study we found that H5N1 viruses intranasally inoculated into cattle do not migrate to the mammary glands. We demonstrated that H5N1 virus replicated in the mouths of calves can be transmitted to the mammary glands of lactating cattle during sucking, thus revealing how the H5N1 virus enters the mammary glands of cows in nature. Cross-nursing (also termed intersucking) in lactating cattle has been reported to occur in 1%–50% of dairy farms and in 0.5%–40% of the dairy cows on those farms [20–26]. Given that the mammary glands of dairy cattle are highly susceptible to different H5N1 viruses, management of cattle that self-nurse or cross-nurse will be very important for preventing the H5N1 virus from invading the mammary glands of cows.

Binding to sialic acid receptors is the first step for influenza virus to infect cells. Lakdawala *et al.* reported that avian-type receptor-binding H1N1 virus easily obtains mutations conferring human-type receptor binding capability during its replication in the soft palate of ferrets, which express human-type sialic acid receptors [38]. Eisfeld *et al.* and Song *et al.* observed that the cattle H5N1 virus bound weakly to human-type receptors [18,39]; however, we and others found that the currently circulating clade 2.3.4.4b H5N1 viruses exclusively bind to avian-type receptors (Fig. S13) [40,41]. The high level of avian-type receptors in the oral tissues of cattle support infection by H5N1 virus via contaminated feed or water. Human-type sialic acid receptors are also widely detected in the oral tissues, teats and mammary glands of cattle (Fig. 3a and Fig. S6) [42–44]. Such receptor properties of cattle may favor the H5N1 virus acquiring mutations to bind to human-type receptors and increase its pandemic potential [45–48]. Therefore, careful monitoring of the mutations of cattle viruses is important for H5N1 influenza pandemic preparedness.

Laboratory studies and epidemiology information from cattle farms in the USA indicate that different genotypes of the H5N1 virus, not just genotype B3.13, may pose a threat to the dairy industry [49]. We detected amino acid substitutions with a low ratio at different positions in six genes and a high ratio of M595I in the PA of the TS/23 virus recovered from cattle (Table S8). It remains to be investigated how these changes affect the biological properties of the TS/23 virus. Dholakia *et al.* suggested that PB2-M631L is a cattle-adapted substitution of the B3.13 H5N1 cattle virus [50], Halwe *et al.* detected the PB2-E627K substitution in the euDG genotype H5N1 virus recovered from cattle [8], and studies indicate that these substitutions increase the polymerase activities of avian influenza viruses in mammalian cells [51–58], thereby increasing their zoonotic spillover risk. Therefore, control of H5N1 virus infection in cattle is important for both animal and human health. Since inactivated and DNA vaccines bearing HA derived from the clade 2.3.4.4b virus could not only completely prevent the naturally occurring H5N1 virus infection in cattle, but also prevent mammary gland infection caused by high-dose virus inoculation that may occur during milking, we speculate that other types of vaccines, such as subunit vaccines or mRNA vaccines based on the 2.3.4.4b HA gene, may also provide similar protection. Vaccination would be an effective and economical strategy to stop the ongoing H5N1 virus transmission in cattle and eliminate the threat it poses to public health.

## MATERIALS AND METHODS

All experiments with live H5N1 viruses were conducted in Biosafety Level 3 (BSL-3) and Animal Biosafety Level 3+ (ABSL-3+) laboratory facilities at the Harbin Veterinary Research Institute (HVRI), Chinese Academy of Agricultural Sciences (CAAS), which are approved for such use by the Ministry of Agriculture and the China National Accreditation Service for Conformity Assessment. The ethical protocols were reviewed and approved by the Committee on the Ethics of Animal Experiments at HVRI, CAAS, under approval number 240531-01-GJ for dairy cattle.

Detailed descriptions of materials and methods are available as supplementary data at *NSR* online.

## Supplementary Material

nwaf262_Supplemental_Files

## Data Availability

All data are available in the manuscript or the supplementary materials. Sequences of the viruses used in this study are in the GISAlD database (TS/23: EPI_ISL_19616578, DK/24: EPI_ISL_19616579).

## References

[1] Hu X, Saxena A, Magstadt DR et al. Genomic characterization of highly pathogenic avian influenza A H5N1 virus newly emerged in dairy cattle. Emerg Microbes Infect 2024; 13: 2380421. 10.1080/22221751.2024.2380421 39008278 PMC11271078

[2] Mostafa A, Naguib MM, Nogales A et al. Avian influenza A (H5N1) virus in dairy cattle: origin, evolution, and cross-species transmission. mBio 2024; 15: e0254224. 10.1128/mbio.02542-24 39535188 PMC11633217

[3] Oguzie JU, Marushchak LV, Shittu I et al. Avian influenza A(H5N1) virus among dairy cattle, Texas, USA. Emerg Infect Dis 2024; 30: 1425–9. 10.3201/eid3007.240717 38848249 PMC11210641

[4] Singh G, Trujillo JD, McDowell CD et al. Detection and characterization of H5N1 HPAIV in environmental samples from a dairy farm. Virus Genes 2024; 60: 517–27. 10.1007/s11262-024-02085-4 39008139 PMC11686969

[5] Baker AL, Arruda B, Palmer MV et al. Dairy cows inoculated with highly pathogenic avian influenza virus H5N1. Nature 2025; 637: 913–20. 10.1038/s41586-024-08166-6 39406346 PMC11754099

[6] Burrough ER, Magstadt DR, Petersen B et al. Highly pathogenic avian influenza A(H5N1) clade 2.3.4.4b virus infection in domestic dairy cattle and cats, United States, 2024. Emerg Infect Dis 2024; 30: 1335–43. 10.3201/eid3007.240508 38683888 PMC11210653

[7] Caserta LC, Frye EA, Butt SL et al. Spillover of highly pathogenic avian influenza H5N1 virus to dairy cattle. Nature 2024; 634: 669–76. 10.1038/s41586-024-07849-4 39053575 PMC11485258

[8] Halwe NJ, Cool K, Breithaupt A et al. H5N1 clade 2.3.4.4b dynamics in experimentally infected calves and cows. Nature 2025; 637: 903–12. 10.1038/s41586-024-08063-y 39321846 PMC11754106

[9] Centers for Disease Control and Prevention . H5 Bird Flu: Current Situation. U.S. Centers for Disease Control and Prevention, Atlanta, Georgia, U.S. 2025. https://www.cdc.gov/bird-flu/situation-summary/?CDC_AAref_Val=https://www.cdc.gov/flu/avianflu/avian-flu-summary.htm (29 June 2025, date last accessed).

[10] Le Sage V, Campbell AJ, Reed DS et al. Persistence of influenza H5N1 and H1N1 viruses in unpasteurized milk on milking unit surfaces. Emerg Infect Dis 2024; 30: 1721–3. 10.3201/eid3008.240775 38914418 PMC11286056

[11] Neumann G, Kawaoka Y. Highly pathogenic H5N1 avian influenza virus outbreak in cattle: the knowns and unknowns. Nat Rev Micro 2024; 22: 525–6. 10.1038/s41579-024-01087-1 PMC12720498 39060613

[12] Science . Bird flu may be spreading in cows via milking and herd transport. News in Science 2025. https://www.science.org/content/article/bird-flu-may-be-spreading-cows-milking-and-herd-transport (29 June 2025, date last accessed).

[13] U.S. Department of Agriculture . Highly Pathogenic Avian Influenza (HPAI) H5N1 Detections in Alpacas. U.S. Department of Agriculture, Washington, DC, U.S. 2025. https://www.aphis.usda.gov/livestock-poultry-disease/avian/avian-influenza/hpai-detections/mammals/highly-pathogenic-avian (29 June 2025, date last accessed).

[14] Science . U.S. Dairy Farm Worker Infected As Bird Flu Spreads to Cows in Five States. News in Science 2025. https://www.science.org/content/article/us-dairy-farm-worker-infected-as-bird-flu-spreads-to-cows-in-five-states#:∼:text=Texas%20officials%20today%20issued%20a,influenza%20viruses%20jump%20into%20humans (29 June 2025, date last accessed).

[15] U.S. Department of Agriculture . Detections of Highly Pathogenic Avian Influenza in Mammals. U.S. Department of Agriculture, Washington, DC, U.S. 2025. https://www.aphis.usda.gov/livestock-poultry-disease/avian/avian-influenza/hpai-detections/mammals (29 June 2025, date last accessed).

[16] Peacock TP, Moncla L, Dudas G et al. The global H5N1 influenza panzootic in mammals. Nature 2025; 637: 304–13. 10.1038/s41586-024-08054-z 39317240

[17] Elsmo EJ, Wünschmann A, Beckmen KB et al. Highly pathogenic avian influenza A(H5N1) virus clade 2.3.4.4b infections in wild terrestrial mammals, United States, 2022. Emerg Infect Dis 2023; 29: 2451–60. 10.3201/eid2912.230464 37987580 PMC10683806

[18] Eisfeld AJ, Biswas A, Guan L et al. Pathogenicity and transmissibility of bovine H5N1 influenza virus. Nature 2024; 633: 426–32. 10.1038/s41586-024-07766-6 38977017 PMC11390473

[19] Gu C, Maemura T, Guan L et al. A human isolate of bovine H5N1 is transmissible and lethal in animal models. Nature 2024; 636: 711–8. 10.1038/s41586-024-08254-7 39467571 PMC12629513

[20] Keil NM, Audigé L, Langhans W. Is intersucking in dairy cows the continuation of a habit developed in early life? J Dairy Sci 2001; 84: 140–6. 10.3168/jds.S0022-0302(01)74462-1 11210026

[21] Lidfors L, Isberg L. Intersucking in dairy cattle—review and questionnaire. Appl Anim Behav Sci 2003; 80: 207–31. 10.1016/S0168-1591(02)00215-0

[22] Mahmoud ME, Mahmoud FA, Ahmed AE. Impacts of self- and cross-sucking on cattle health and performance. Vet World 2016; 9: 922–8. 10.14202/vetworld.2016.922-928 27733790 PMC5057028

[23] Wood PD, Smith GF, Lisle MF. A survey of intersucking in dairy herds in England and Wales. Vet Rec 1967; 81: 396–8. 10.1136/vr.81.16.396 6069564

[24] Peterse DJ, Rutgers B, Schaftenaar W et al. [Studies on intersucking in dairy cattle (author's transl)]. Tijdschr Diergeneeskd 1978; 103: 485–9. 565958

[25] El-Sherif MW, Saber MS, Abd Elkawy MA et al. Effectiveness of surgical interventions for self-sucking in dairy cattle: a comparative study. SVU-Int J Vet Sci 2023; 6: 130–7. 10.21608/svu.2023.220754.1280

[26] Salman Y, Semieka M, Karmi M et al. A novel surgical technique for prevention of self-sucking in cattle and buffaloes: tongue piercing. BMC Vet Res 2022; 18: 192. 10.1186/s12917-022-03283-8 35596220 PMC9121546

[27] Shi J, Zeng X, Cui P et al. Alarming situation of emerging H5 and H7 avian influenza and effective control strategies. Emerg Microbes Infect 2023; 12: 2155072. 10.1080/22221751.2022.2155072 36458831 PMC9754034

[28] Zeng X, Tian G, Shi J et al. Vaccination of poultry successfully eliminated human infection with H7N9 virus in China. Sci China Life Sci 2018; 61: 1465–73. 10.1007/s11427-018-9420-1 30414008

[29] Shi J, Deng G, Ma S et al. Rapid evolution of H7N9 highly pathogenic viruses that emerged in China in 2017. Cell Host Microbe 2018; 24: 558–68. 30269969 10.1016/j.chom.2018.08.006 PMC6310233

[30] Li C, Bu Z, Chen H. Avian influenza vaccines against H5N1 ‘bird flu’. Trends Biotechnol 2014; 32: 147–56. 10.1016/j.tibtech.2014.01.001 24491922

[31] Li C, Chen H. H7N9 influenza virus in China. Cold Spring Harb Perspect Med 2021; 11: a038349. 10.1101/cshperspect.a038349 32205415 PMC8327827

[32] Cui P, Shi J, Wang C et al. Global dissemination of H5N1 influenza viruses bearing the clade 2.3.4.4b HA gene and biologic analysis of the ones detected in China. Emerg Microbes Infect 2022; 11: 1693–704. 10.1080/22221751.2022.2088407 35699072 PMC9246030

[33] Gu W, Shi J, Cui P et al. Novel H5N6 reassortants bearing the clade 2.3.4.4b HA gene of H5N8 virus have been detected in poultry and caused multiple human infections in China. Emerg Microbes Infect 2022; 11: 1174–85. 10.1080/22221751.2022.2063076 35380505 PMC9126593

[34] Zeng X, He X, Meng F et al. Protective efficacy of an H5/H7 trivalent inactivated vaccine (H5-Re13, H5-Re14, and H7-Re4 strains) in chickens, ducks, and geese against newly detected H5N1, H5N6, H5N8, and H7N9 viruses. J Integr Agric 2022; 21: 2086–94. 10.1016/S2095-3119(22)63904-2

[35] Cui P, Zeng X, Li X et al. Genetic and biological characteristics of the globally circulating H5N8 avian influenza viruses and the protective efficacy offered by the poultry vaccine currently used in China. Sci China Life Sci 2022; 65: 795–808. 10.1007/s11427-021-2025-y 34757542

[36] Campbell AJ, Brizuela K, Lakdawala SS. mGem: transmission and exposure risks of dairy cow H5N1 influenza virus. mBio 2025; 16: e0294424. 10.1128/mbio.02944-24 39932310 PMC11898566

[37] Krämer K . Daily briefing: evidence grows that cows spread bird flu through their milk. Nature 2024; doi: 10.1038/d41586-024-01709-x. 38849473

[38] Lakdawala SS, Jayaraman A, Halpin RA et al. The soft palate is an important site of adaptation for transmissible influenza viruses. Nature 2015; 526: 122–5. 10.1038/nature15379 26416728 PMC4592815

[39] Song H, Hao T, Han P et al. Receptor binding, structure, and tissue tropism of cattle-infecting H5N1 avian influenza virus hemagglutinin. Cell 2025; 188: 919–29. 10.1016/j.cell.2025.01.019 39848246

[40] Chopra P, Ray SD, Page CK et al. Receptor-binding specificity of a bovine influenza A virus. Nature 2025; 640: E21–7. 10.1038/s41586-025-08822-5 40240861 PMC13280417

[41] Santos JJS, Wang S, McBride R et al. Bovine H5N1 binds poorly to human-type sialic acid receptors. Nature 2025; 640: E18–20. 10.1038/s41586-025-08821-6 40240859

[42] Kristensen C, Jensen HE, Trebbien R et al. Avian and human influenza A virus receptors in bovine mammary gland. Emerg Infect Dis 2024; 30: 1907–11. 10.3201/eid3009.240696 39127127 PMC11347012

[43] Nelli RK, Harm TA, Siepker C et al. Sialic acid receptor specificity in mammary gland of dairy cattle infected with highly pathogenic avian influenza A(H5N1) virus. Emerg Infect Dis 2024; 30: 1361–73. 10.3201/eid3007.240689 38861554 PMC11210646

[44] Rios Carrasco M, Grone A, van den Brand JMA et al. The mammary glands of cows abundantly display receptors for circulating avian H5 viruses. J Virol 2024; 98: e0105224. 10.1128/jvi.01052-24 39387556 PMC11575340

[45] Lin TH, Zhu X, Wang S et al. A single mutation in bovine influenza H5N1 hemagglutinin switches specificity to human receptors. Science 2024; 386: 1128–34. 10.1126/science.adt0180 39636969 PMC12633761

[46] Imai M, Watanabe T, Hatta M et al. Experimental adaptation of an influenza H5 HA confers respiratory droplet transmission to a reassortant H5 HA/H1N1 virus in ferrets. Nature 2012; 486: 420–8. 10.1038/nature10831 22722205 PMC3388103

[47] Herfst S, Schrauwen EJ, Linster M et al. Airborne transmission of influenza A/H5N1 virus between ferrets. Science 2012; 336: 1534–41. 10.1126/science.1213362 22723413 PMC4810786

[48] Gao Y, Zhang Y, Shinya K et al. Identification of amino acids in HA and PB2 critical for the transmission of H5N1 avian influenza viruses in a mammalian host. PLoS Pathog 2009; 5: e1000709. 10.1371/journal.ppat.1000709 20041223 PMC2791199

[49] U.S. Department of Agriculture . APHIS confirms D1.1 genotype in dairy cattle in Nevada. U.S. Department of Agriculture, Washington, DC, U.S. 2025. https://www.aphis.usda.gov/news/program-update/aphis-confirms-d11-genotype-dairy-cattle-nevada-0 (29 June 2025, date last accessed).

[50] Dholakia V, Quantrill JL, Richardson S et al. Polymerase mutations underlie early adaptation of H5N1 influenza virus to dairy cattle and other mammals. bioRxiv 2025;. 10.1101/2025.01.06.631435 PMC12905184 41545362

[51] Hatta M, Gao P, Halfmann P et al. Molecular basis for high virulence of Hong Kong H5N1 influenza A viruses. Science 2001; 293: 1840–2. 10.1126/science.1062882 11546875

[52] Liang L, Jiang L, Li J et al. Low polymerase activity attributed to PA drives the acquisition of the PB2 E627K mutation of H7N9 avian influenza virus in mammals. mBio 2019; 10:10.1128/mbio.01162-19. PMC6581862 31213560

[53] Shi J, Deng G, Kong H et al. H7N9 virulent mutants detected in chickens in China pose an increased threat to humans. Cell Res 2017; 27: 1409–21. 10.1038/cr.2017.129 29151586 PMC5717404

[54] Subbarao EK, Kawaoka Y, Murphy BR. Rescue of an influenza A virus wild-type PB2 gene and a mutant derivative bearing a site-specific temperature-sensitive and attenuating mutation. J Virol 1993; 67: 7223–8. 10.1128/jvi.67.12.7223-7228.1993 8230444 PMC238184

[55] Kong H, Ma S, Wang J et al. Identification of key amino acids in the PB2 and M1 proteins of H7N9 influenza virus that affect its transmission in guinea pigs. J Virol 2019; 94: 10.1128/JVI.01180-19 PMC6912098 31597771

[56] Li J, Deng G, Shi J et al. Genetic and biological characterization of H3N2 avian influenza viruses isolated from poultry farms in China between 2019 and 2021. Transbound Emerg Dis 2023; 2023: 8834913. 10.1155/2023/8834913 40303673 PMC12016730

[57] Zhang H, Li X, Guo J et al. The PB2 E627K mutation contributes to the high polymerase activity and enhanced replication of H7N9 influenza virus. J Gen Virol 2014; 95: 779–86. 10.1099/vir.0.061721-0 24394699

[58] Li X, Shi J, Guo J et al. Genetics, receptor binding property, and transmissibility in mammals of naturally isolated H9N2 avian influenza viruses. PLoS Pathog 2014; 10: e1004508. 10.1371/journal.ppat.1004508 25411973 PMC4239090

