# Supplementary material for: H5N1 virus invades the mammary glands of dairy cattle through ‘mouth-to-teat’ transmission
Source: Natl Sci Rev. 2025 Jul 1;12(9):nwaf262. doi: 10.1093/nsr/nwaf262 (PMC12342610; doi:10.1093/nsr/nwaf262)
Supplement: nwaf262_Supplemental_Files [file nwaf262_supplemental_files.zip › Shi_Fig_S1.pdf]

a

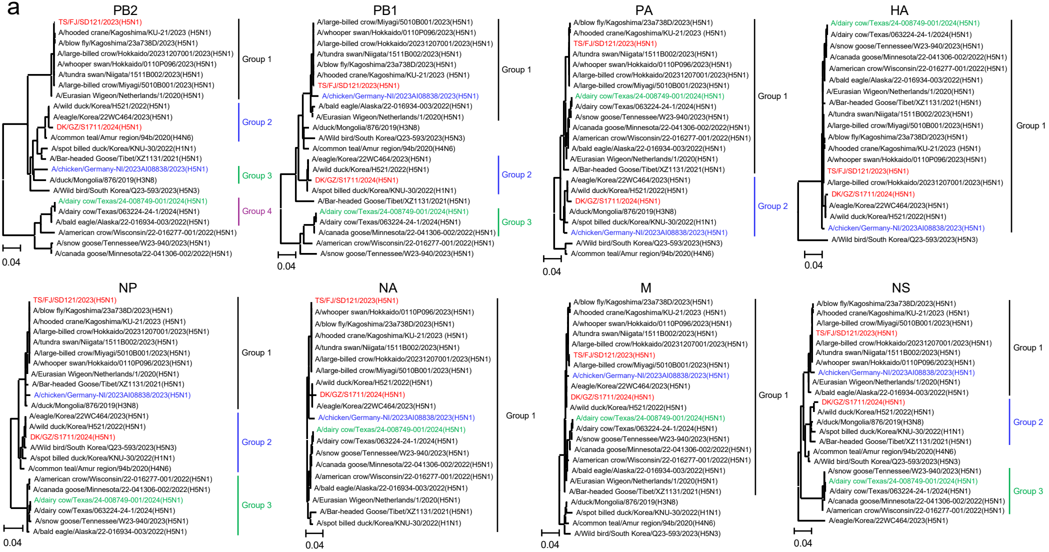

b

Virus	Group of each gene segment in the phylogenetic tree								Genotype
	PB2	PB1	PA	HA	NP	NA	M	NS	
TS/FJ/SD121/2023(H5N1)	**1**	**1**	**1**	**1**	**1**	**1**	**1**	**1**	G1
A/chicken/Germany-NI/2023AI08838/2023(H5N1)	**3**	**1**	**2**	**1**	**1**	**1**	**1**	**1**	G2 (EuDG)
A/dairy cow/Texas/24-008749-001/2024(H5N1)	**4**	**3**	**1**	**1**	**3**	**1**	**1**	**3**	G3 (B3.13)
DK/GZ/S1711/2024(H5N1)	**2**	**2**	**2**	**1**	**2**	**1**	**1**	**2**	G4
