# Supplementary material for: H5N1 virus invades the mammary glands of dairy cattle through ‘mouth-to-teat’ transmission
Source: Natl Sci Rev. 2025 Jul 1;12(9):nwaf262. doi: 10.1093/nsr/nwaf262 (PMC12342610; doi:10.1093/nsr/nwaf262)
Supplement: nwaf262_Supplemental_Files [file nwaf262_supplemental_files.zip › Shi_Fig_S8.pdf]

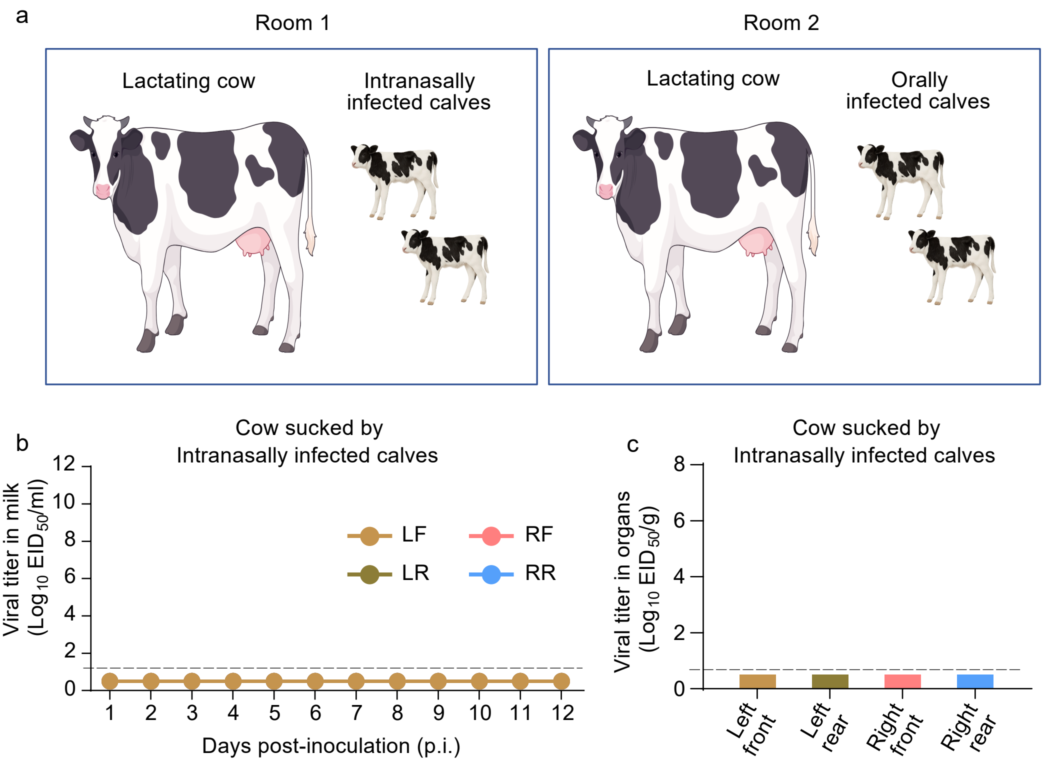
a
Room 1
Room 2
Lactating cow
Intranasally infected calves
Lactating cow
Orally infected calves
b
Cow sucked by Intranasally infected calves
Viral titer in milk (Log_{10} EID_{50}/ml)
0
2
4
6
8
10
12
LF
RF
LR
RR
1
2
3
4
5
6
7
8
9
10
11
12
Days post-inoculation (p.i.)
c
Cow sucked by Intranasally infected calves
Viral titer in organs (Log_{10} EID_{50}/g)
0
2
4
6
8
Left front
Left rear
Right front
Right rear
