# Supplementary material for: H5N1 virus invades the mammary glands of dairy cattle through ‘mouth-to-teat’ transmission
Source: Natl Sci Rev. 2025 Jul 1;12(9):nwaf262. doi: 10.1093/nsr/nwaf262 (PMC12342610; doi:10.1093/nsr/nwaf262)
Supplement: nwaf262_Supplemental_Files [file nwaf262_supplemental_files.zip › Shi_supplemental_manuscript.docx]

Supplementary information

**H5N1 virus invades the mammary glands of dairy cattle through “mouth-to-teat” transmission**

Jianzhong Shi, Huihui Kong, Pengfei Cui, Guohua Deng, Xianying Zeng, Yongping Jiang, Xijun He, Xianfeng Zhang, Lei Chen, Yichao Zhuang, Yan Wang, Jinming Ma, Jiaqi Li, Yaping Zhang, Congcong Wang, Chen He, Jiaxin Yang, Jiongjie Li, Wenyu Liu, Jinyu Yang, Shenggang Mao, Jinxiong Liu, Pucheng Chen, Guobin Tian, Chengjun Li, Yuntao Guan, Zhigao Bu, and Hualan Chen^*^

^*^Corresponding author: chenhualan@caas.cn

**The PDF file includes:**

Materials and Methods

Supplementary Text

Supplementary Data Figures S1 to S13

Supplementary Data Tables S1 to S8

**Materials and Methods**

**Facility and ethics statements**

All experiments with live H5N1 viruses were conducted in Biosafety Level 3 (BSL-3) and Animal Biosafety Level 3 (ABSL-3) laboratory facilities at the Harbin Veterinary Research Institute (HVRI), Chinese Academy of Agricultural Sciences (CAAS), which are approved for such use by the Ministry of Agriculture and the China National Accreditation Service for Conformity Assessment. The ethical protocols were reviewed and approved by the Committee on the Ethics of Animal Experiments at HVRI, CAAS, under approval number 240531-01-GJ for dairy cattle.

**Viruses**

Two avian H5N1 strains, A/tundra swan/Fujian/SD121/2023 (TS/23) (GISAID accession no. EPI_ISL_19616578) and A/duck/Guizhou/S1711/2024 (DK/24) (GISAID accession no. EPI_ISL_19616579), were isolated from the organs of a dead tundra swan found in a wetland in Fujian Province, China, and from duck swabs collected at a live poultry market in China, respectively. The dairy cow H5N1 strain, A/dairy cow/Texas/24-008749-001/2024 (DC/24), the first H5N1 strain isolated from a dairy cow in Texas, USA, was generated using sequence data available in the database (GISAID accession no. EPI_ISL_19014384), as previously described [59]. These viruses were propagated in 10-day-old specific pathogen-free chicken embryonated eggs, and allantoic fluids were harvested 48 h after incubation at 37°C to prepare virus stocks. The viral dose was titrated in embryonated chicken eggs, with the 50% egg infectious dose (EID_50_) determined using the Reed and Muench method.

**Cells**

Madin-Darby canine kidney (MDCK) cells and human embryonic kidney 293T cells were obtained from the American Type Culture Collection (ATCC). MDCK cells were cultured in Dulbecco's Modified Eagle Medium (DMEM) supplemented with 5% newborn calf serum (NCS), while 293T cells were maintained in DMEM containing 10% fetal bovine serum (FBS). Both cell lines were incubated at 37°C in 5% CO₂.

**Preparation of the inactivated vaccine**

The oil-emulsion inactivated vaccine was prepared by using the H5-Re14 seed virus at Harbin Weike Biotechnology Co., Ltd. (Harbin, China). The H5-Re14 seed virus bearing the hemagglutinin (HA) and neuraminidase (NA) genes from a clade 2.3.4.4b virus, A/whooper swan/Shanxi/4-1/2020 (H5N8) (WS/SX/4-1/20), and six internal genes from A/Puerto Rico/8/1934 (H1N1) (PR8), was generated through reverse genetics in a previous study[34]. The antigen was propagated by growing the H5-Re14 virus in 10-day-old embryonated chicken eggs and adjusted to 1024 HA units/0.1 ml. After inactivation with 0.2% formalin, the antigen and Montanide™ ISA 71 VG adjuvant (Seppic, Paris, France) were emulsified to create an oil-in-water emulsion; the ratio of the antigen to adjuvant was 1:1.8.

**Preparation of the DNA vaccine**

The H5 DNA vaccine used in this study was a laboratory-prepared DNA-lipid nanoparticle vaccine. The DNA plasmid was constructed with a strategy similar to that reported by Jiang et al. [60]. The insert was a chicken codon-optimized HA gene, which is the same as a cattle codon-optimized HA gene, encoding a similar amino acid sequence to that of the HA of the clade 2.3.4.4 H5N8 virus WS/SX/4-1/20 virus[35]. The DNA plasmids were extracted from bacteria by using the EndoFree Plasmid Maxi Kit (QIAGEN) and dissolved in 50 mM citrate buffer (pH 4.0) to a concentration of 150 μg/ml. To generate the DNA-lipid nanoparticles, lipid adjuvant, which contains ionizable lipid, DSPC (1,2-Distearoyl-sn-glycero-3-phosphocholine), cholesterol, and PEGylated lipid in ethanol with molar ratios of 100:17:80:3, was mixed with the plasmid DNA at a 1:3 (volume:volume) ratio using a microfluidic mixer (INano E, Micro&Nano Technology Inc, China) (INano E, Micro&Nano, China). The DNA-lipid nanoparticles were diluted with sterile Ca^2+-^Mg^2+-^free PBS and concentrated using Amicon® Ultra-15 centrifugal filters (30 kDa MWCO, Millipore). The DNA-lipid nanoparticles were sterilized with a 0.22-μm filter, and the final preparation containing 125 μg/ml DNA in PBS was stored at 4℃ until use.

**Phylogenetic analysis**

The phylogenetic trees were generated with the Molecular Evolutionary Genetics Analysis (MEGA) 11 software package with the neighbor-joining method, and 96% sequence identity cutoffs were used to categorize the gene groups in the phylogenetic trees.

**Receptor-binding preference analysis**

The receptor-binding specificity of influenza virus was analyzed by using biolayer interferometry, with binding affinity measured on the Octet Red 96 system (FortéBio, United States), as previously described [61,62]. Two types of biotinylated glycans, Neu5Ac (α2-3)Gal (β1-4)GlcNAc (3′SLN) and Neu5Ac (α2-6)Gal (β1-4)GlcNAc (6′SLN), were used in this study. Streptavidin sensors were first loaded with 2.5 μg/ml glycan until responses reached 0.5 nm for 3′SLN and 6′SLN. The sensors were then incubated with a sample containing 128 HA units of the test virus in HBS-EP buffer (0.005% surfactant P20, 150 mM NaCl, 10 mM HEPES, pH 7.4, 3 mM EDTA). To prevent interference from neuraminidase activity, 10 mM oseltamivir was added to the buffer. The binding interaction was monitored at 30°C for 500 seconds.

**Virus replication in dairy cattle**

Fifteen 3–6-year-old lactating Holstein cows, obtained from a local dairy farm, were used for H5N1 virus replication evaluation. The cows were housed in ABSL-3 rooms for three days to acclimate to their environment before the experiments were performed. Each cow was placed in a separate stall (1.8 m × 2.5 m), and its body temperature and milk color were monitored every day. Throughout the experimental period, the cows were milked twice daily using a milking machine. A 5-ml syringe with a straight oral gavage needle (16G × 110 mm) was used to deliver the virus. For nasal inoculation, groups of three cows were first anesthetized with Xylazine (0.2 mg/kg, intramuscular injection) and then inoculated intranasally with 2 × 10⁶ EID_50_ of H5N1 virus in a total volume of 2 ml (1 ml per nostril). For intramammary gland inoculation, the teats of cows were first disinfected with an alcohol-based disinfectant. The left front and right rear mammary glands were then inoculated with 200 EID_50_ of H5N1 virus in a total volume of 2 mL (1 ml per gland). Nasal swabs, oral swabs, rectal swabs, and milk from each udder were collected daily, beginning at day 1 post-infection (p.i.) to monitor virus shedding. One cow from each group was euthanized on days 3, 6, and 14 p.i. respectively, and their tissues, including nasal turbinate, soft palate, root of the tongue, larynx, tonsils, sublingual gland, parotid gland, submandibular gland, submandibular lymph nodes, trachea, bronchus, six lung lobes, heart, liver, spleen, pancreas, kidney, brain, esophagus, four mammary glands, rumen, reticulum, omasum, abomasum, duodenum, jejunum, ileum, cecum, colon, rectum, uterus, ovaries, bladder, thymus gland, and blood were collected for virus titration in eggs.

To collect nasal swabs, a sterile cotton swab (~20 cm) was inserted into each nostril of the cattle and gently rotated five times. To collect oral swabs, two swabs were gently inserted into the oral cavity and firmly rubbed or rotated against the inner surfaces of the cheeks, gums, and tongue. To collect rectal swabs, two sterile cotton swabs were inserted into the rectum and gently rotated five times. Immediately after sampling, swabs from the same source of one animal were placed into a 15-ml centrifuge tube containing PBS (1 ml of PBS/swab) with 2,000 U/mL penicillin and 2,000 µg/mL streptomycin and vortexed for 30 s. The liquid was then harvested for virus titration. To collect organ and tissue samples, each tissue was individually harvested using a sterile pair of scissors and tweezers to prevent cross-contamination. Briefly, each organ or tissue sample was divided into five equally sized segments, and a small portion from each segment was collected and added to a single sterile 2-mL centrifuge tube. Prior to virus titration, the collected tissues were weighed and mixed with PBS containing 2,000 U/mL penicillin and 2,000 µg/mL streptomycin at a ratio of 1 g of tissue to 1 mL of PBS. Samples were then homogenized using a frozen tissue homogenizer, and the supernatants were collected and 10-fold serially diluted for virus titration in 10-day-old embryonated chicken eggs. Virus titers were subsequently measured using a hemagglutination assay with 1% chicken red blood cells (cRBCs).

**Next generation sequencing**

The complete genomes of the H5N1 viruses were sequenced using 200-cycle, 2×100 bp paired-end libraries on the MGISEQ-200 platform (MGI, Shenzhen, China), based on DNA nanoball (DNB) and combinatorial Probe-Anchor Synthesis (cPAS) technologies. Sequencing was performed at the Harbin Veterinary Research Institute, Chinese Academy of Agricultural Sciences. Raw reads were processed with SOAPnuke (v2.1.9) to remove adapters and low-quality sequences. Clean reads were aligned to the reference genome using HISAT2 (v2.2.1). Unmapped reads, potentially representing strain-specific sequences, were extracted with Samtools (v1.20) and filtered for rRNA using SortMeRNA (v4.3.7). The remaining reads were assembled de novo with MEGAHIT (v1.2.9) under default parameters.

**Detection of sialic acid receptor expression in tissues**

We investigated the expression of sialic acid receptors in different tissues of cows, including the nasal turbinate, soft palate, tonsils, root of the tongue, sublingual gland, submandibular gland gland, larynx, parotid gland, teats, trachea, lungs, and mammary glands. The tissues were fixed in 10% neutral buffered formalin for 3 days before they were embedded in paraffin. Then, 4-µm thick sections were deparaffinized and rehydrated. After this, the slides were blocked with 5% bovine serum albumin for 1 h, followed by three washes with PBS. The sections were then incubated with three fluorescein-labeled lectins: Sambucus nigra lectin (SNA), Maackia Amurensis Lectin I (MAL-I), or Maackia Amurensis Lectin II (MAL-II) (Vector Laboratories). SNA specifically binds to α2,6-linked SAs (human-type receptor), MAL-I specifically binds to N-linked or O-linked glycans containing α2,3-gal-β (1-4) GlcNAc (avian-type receptor), while MAL-II binds to O-linked glycans with α2,3-gal-β (1-3) GalNAc (avian-type receptor) [63]. After incubating at room temperature for 2 h, the fluorescence was observed under a microscope.

**“Mouth-to-teat” transmission study**

To test whether the H5N1 virus in the cattle oral cavity could be transmitted to the mammary glands of cattle during the behavior of self-sucking or cross-sucking, we infected a pair of calves with 10^6^EID_50_ of the DC/24 virus intranasally and another pair orally. Six hours later each pair of calves was housed with a lactating cow in one room as shown in Fig. S8A, and the calves were allowed to suck the teats of the lactating cattle as frequently as needed. The nasal and oral swabs from the calves and the milk from the lactating cattle were collected every day for 12 days for virus titration in eggs. We also collected tissues from the mammary glands of lactating cows that were euthanized at the end of observation to test the virus titers in eggs. Body temperature and the milk changes were monitored daily.

**Vaccination and challenge study in dairy cattle**

Groups of six dairy cattle were intramuscularly immunized with two doses in 4 ml of either an inactivated vaccine or a DNA vaccine at an interval of 3 weeks. Vaccines were injected at two sites (2 ml at each site) on the dorsal side of the cattle's neck muscle. Six cows were not vaccinated and used as controls. Blood and milk samples were collected weekly to assess the antibody response. Due to the limitations of laboratory space, different viruses were challenged at different time points. Two weeks after the second dose, three cattle from each group were transferred to the ABSL-3 facility and challenged with the TS/23 virus by intranasal and intramammary gland inoculation simultaneously, using the same dose as was used for the infection study described above. The three remaining cattle from each group were challenged similarly with the DC/24 virus five weeks after the second dose. One cattle from each group was euthanized on days 3, 6, and 12 post-challenge (p.c.), respectively. Nasal swabs, oral swabs, and milk were collected daily from all available animals to monitor virus shedding. Tissues from the oral cavity, respiratory tract, and mammary glands were collected from euthanized animals for virus titration in eggs, and lungs and mammary glands were also collected for histological study.

To investigate whether the vaccines could prevent virus replication after high-dose intramammary gland inoculation, groups of three dairy cattle were intramuscularly immunized with two doses in 4 ml of either an inactivated vaccine or a DNA vaccine at an interval of 3 weeks. Three unvaccinated cattle served as controls. Blood and milk samples were collected weekly to assess antibody responses. Two weeks after the second dose, the cattle were transferred to the ABSL-3 facility and challenged with DC/24 virus via intramammary gland inoculation: 10^2^ EID_50_ in 1 ml to the left rear teat, 10^4^ EID_50_ in 1 ml to the right front teat and 10^6^ EID_50_ in 1 ml to the right rear teat. Milk from each udder of the cattle was sampled daily for virus titration in eggs. One animal from each group was euthanized on days 3, 6, and 12 p.c., respectively, and tissues from their intramammary glands were collected for virus titration in eggs.

**Antibody detection**

To investigate the antibody response of cattle following vaccine inoculation, serum and milk samples were collected from vaccinated and control dairy cattle weekly, the hemagglutinin inhibition (HI) antibody levels in serum and neutralization antibody levels in milk were evaluated by using the H5-Re14 vaccine seed virus. Antibodies against the challenge virus were also evaluated in the samples collected at the time point before challenge.

For the HI assay, 25 μl of two-fold serially diluted serum was prepared across the rows of a V-bottom 96-well plate. Then, 25 μL of the test virus, containing 4 HA units was added to each well. After a 30-minute incubation at room temperature, 25 μl of 1% cRBCs was added to each well, followed by an additional 30 minutes of incubation. The HI titer was defined as the highest dilution of serum that completely inhibited cRBC agglutination.

For the micro-neutralization assay, milk was centrifuged at 1,200 rpm for 2 minutes, then 50 μl of two-fold serially diluted milk was prepared across the rows of a V-bottom 96-well plate. Next, 50 μl of test virus, containing 100 EID_50_ of virus, was added to each well and incubated for 1 h at 37°C. The mixture was then transferred to a 96-well plate seeded with MDCK cells. After a 1-h incubation, the plate was washed twice with phosphate-buffered saline (PBS) and then refilled with Eagle’s minimal essential medium (MEM) containing 0.5 µg/ml N-p-tosyl-L-phenylalanine chloromethyl ketone (TPCK)-treated trypsin. After 48 h of incubation, a hemagglutination assay was performed to check whether viral replication was inhibited. The neutralizing titer was defined as the highest dilution of milk that completely inhibited virus replication.

**Antibody Isotype Analysis**

Antibody isotype analysis was conducted on cattle serum and milk collected two weeks after the second dose of vaccine by performing an indirect enzyme-linked immunosorbent assay (ELISA). Samples from unvaccinated cattle were used as controls. Briefly, 96-well ELISA plates were coated with 100 µL/well of purified H5-Re14 vaccine virus and incubated overnight at 4°C. After being washed three times with PBS containing 0.05% Tween-20 (PBST), the plates were blocked with 5% milk in PBST for 1 h at 37°C. Serum and milk samples were four-fold serially diluted in PBST and added to the plates (100 µL/well), followed by incubation for 1 h at 37°C. After three washes with PBST, the plates were incubated with sheep anti-bovine IgG or IgA secondary antibodies (Bio-Rad, USA) for 1 h at 37°C. The plates were then incubated with sheep anti-bovine IgG or IgA secondary antibodies (Bio-Rad, USA) for 1 h at 37°C. Finally, the plates were incubated with horseradish peroxidase (HRP)-conjugated anti-sheep IgG antibody (Sigma-Aldrich, USA) for 1 h at 37°C. The reaction was developed using TMB substrate (Thermo Fisher, USA), stopped with 2 M H₂SO₄, and absorbance was measured at 450 nm using a microplate reader.

**Histological study and immunohistochemical analysis**

The lungs and mammary glands of cattle in the intranasal and intramammary glands simultaneously challenged studies were collected for histological study and immunohistochemical analysis. Virus-positive soft palate, tonsil, root of the tongue, and submandibular gland of the cattle infected with the DC/24 virus were used for immunohistochemical analysis. The samples were fixed in 10% neutral-buffered formalin. After fixation for three days, the tissues were embedded in paraffin, and tissue blocks were sectioned into 4-µm slices for hematoxylin-eosin (H&E) staining and immunohistochemical analysis. A mouse monoclonal antibody specific to the influenza NP protein was used as the first antibody, and goat anti-mouse IgG (H+L) Poly-HRP was used as the secondary antibody (ThermoFisher, USA) in the immunohistochemical analysis.

**Supplementary Text**

**Histological study of lung and mammary gland of cattle**

We performed pathological studies on lungs and mammary glands of control and vaccinated cattle that were euthanized on days 3 and 6 post-challenge with different viruses. In the sample from the untreated cattle, no obvious pathological changes were observed in either the lungs (Fig. S11a) or mammary glands (Fig. S11c), and no viral antigen was detected (Fig. S11, b and d). In the lungs collected from control animals that were challenged with the TS/23 virus, significant pathological changes, including extensive alveolar lumen occlusion, characterized by inflammatory cell infiltration and alveolar epithelial cell hyperplasia, were observed (Fig. S11e). In the mammary glands collected from the control animals that were challenged with the TS/23 virus, there was prominent neutrophil infiltration and necrotic debris accumulation in most acini and ducts (Fig. S11g). In the lungs of DC/24-challenged control animals, diffuse and severe alveolar and bronchiolar occlusion, predominantly filled with neutrophils, macrophages, and necrotic debris was exhibited. Necrosis and sloughing of bronchiolar epithelial cells were also evident (Fig. S11m). In the mammary glands of DC/24-challenged control animals, partial destruction of acinar structures was observed, including necrosis and desquamation of acinar epithelial cells within the acinar lumen (Fig. S11o). Viral antigens were detected in the virus-positive lungs (Fig. S11, f and n) and mammary glands (Fig. S11, h and p) of the control cattle challenged with either the TS/23 virus or DC/24 virus. In the vaccinated cattle, neither TS/23 nor DC/24 induced significant pathological changes in the lungs (Fig. S11, i and q) or mammary glands (Fig. S11, k and s), and viral antigens were not detected in any of the lung lobes (Fig. S11, j and r) or mammary glands (Fig. S11, l and t).

**Study limitations**

Since lactating cattle are very sensitive to environmental changes, milk production dropped dramatically when they were transferred to the ABSL-3 facilities; therefore, we were unable to compare the milk production of cattle in different treatment groups.

It was difficult for us to obtain cattle that steal milk in order to reproduce mouth-to-teat transmission in the laboratory setting, because on most dairy farms in China, once a cow is caught stealing milk, it is usually eliminated.

**Supplementary Data Figures S1 to S13**

**Supplementary Figure S1. Phylogenetic analyses of H5N1 viruses.** (**a**) The phylogenetic trees of the PB2, PB1, PA, HA, NP, NA, M, and NS genes. The two Chinese viruses used in this study are shown in red; genotype B3.13 and EuDG virus are in green and blue, receptively. (**b**) Genotypes of the different H5N1 viruses. The group of each gene was defined based on the phylogenetic trees in panel A.

**Supplementary Figure S2. Replication of Eurasia avian H5N1 viruses in dairy cows infected via intranasal inoculation.** Cows were intranasally inoculated with the TS/23 virus (**a**, **e**, and **g**) or the DK/24 virus (**b-d**, **f**, and **h**). Virus in the swabs, milk, and tissues of cows was titrated in eggs. (**a**) Viral titers in rectal swabs of cows infected with the TS/23 virus. Viral titers in nasal swabs (**b**), oral swabs (**c**), and rectal swabs (**d**) of cows infected with the DK/24 virus. Viral titers in milk from different udders of cows infected with the TS/23 virus (**e**) and the DK/24 virus (**f**). Viral titers in the tissues of cows infected with the TS/23 virus (**g**) and the DK/24 virus (**h**). Each color bar represents the value of an individual animal. The horizontal dashed lines in the panels indicate the lower limit of detection.

**Supplementary Figure S3. Amino acid differences between the TS/23 and DK/24 viruses.** The amino acid differences between the two viruses are shown as single letters at the indicated positions. Each amino acid of TK/23 is shown before the number of the position, and each amino acid of DK/24 is shown after the number of the position. The H3 numbering is used for the HA protein.

**Supplementary Figure S4. Replication of H5N1 avian influenza virus in dairy cows infected via intramammary glands inoculation.** Cows were infected with 2×10^2^EID_50_ of TS/23 virus via intramammary glands inoculation, and viral titers in nasal swabs (**a**), oral swabs (**b**), rectal swabs (**c**), and tissues (**d**) of cows wwere titrated in eggs. Each color bar represents the value of an individual animal. The horizontal dashed lines in the panels indicate the lower limit of detection.

**Supplementary Figure S5.** **Replication of DC/24 virus in dairy cows.** Cows were inoculated with the DC/24 virus intranasally (**a-c**) or via the intramammary glands (**d-g**), and virus in the swabs, milk, and tissues of cows was titrated in eggs. Viral titers in rectal swabs (**a**), milk (**b**), and tissues (**c**) of cows infected intranasally with the DC/24 virus. Viral titers in the nasal swabs (**d**), oral swabs (**e**), rectal swabs (**f**), and tissues (**g**) of cows infected through the intramammary glands. Each color bar represents the value of an individual animal. The horizontal dashed lines in the panels indicate the lower limit of detection.

**Supplementary Figure S6.** **Expression of sialic acid receptors in different tissues of dairy cattle.** The tissues were stained with three fluorescein-labeled lectins: Maackia amurensis lectin I (MAL-I) and Maackia amurensis lectin II (MAL-II) stain different avian-type receptors, and Sambucus nigra lectin (SNA) stains human-type receptors. Scale bars = 100 µm.

**Supplementary Figure S7. Unconventional nursing behavior of dairy cattle.** (**a** and **b**) A cow engaged in auto-nursing. (**c**) Cows engaged in mutual-nursing. (**d**) Cows engaged in chain-nursing. Images are royalty-free stock photos.

**Supplementary Figure S8.** Group information of the “mouth-to-teat” transmission **(a**) and viral titers in milk (**b**) and tissues of mammary glands (**c**) of the cow sucked by intranasally infected calves.

**Supplementary Figure S9. Analysis of antibody isotypes induced by different vaccines in cattle.** Levels of IgG (**a** and **b**) and IgA (**c** and **d**) in the serum (**a** and **c**) and milk (**b** and **d**) of vaccinated and control cattle were analyzed by using an ELISA with bovine IgG- and IgA-specific antibodies.

**Supplementary Figure S10. Protective efficacy of different vaccines in cattle against TS/23 virus challenge.** Vaccinated cattle and control cattle were challenged with TS/23 virus through both intranasal and intramammary gland inoculation, and tissues were collected for virus titration in eggs. Viral titers in tissues of cattle euthanized on days 3 (**a**), 6 (**b**), and 12 (**c**) post-challenge (p.c). Each color bar represents the value of an individual animal. The horizontal dashed lines in the panels indicate the lower limit of detection.

**Supplementary Figure S11. Histological lesions of cows.** Pathological studies were performed in the lungs (**a-b, e-f, i-j, m-n,** and **q-r**) and mammary glands (**c-d, g-h, k-l, o-p,** and **s-t**) of control and vaccinated cattle that were euthanized on days 3 and 6 post-challenge with different viruses. Hematoxylin-eosin (H&E) staining in the lungs (**a**) and mammary glands (**c**), and immunohistochemistry in the lungs (**b**) and mammary glands (**d**) of mock cows. H&E staining in the lungs (**e**) and mammary glands (**g**), and immunohistochemistry in the lungs (**f**) and mammary glands (**h**) of control cows that were challenged with the TS/23 virus. H&E staining in the lungs (**i**) and mammary glands (**k**), and immunohistochemistry in the lungs (**j**) and mammary glands (**l**) of vaccinated cows that were challenged with the TS/23 virus. H&E staining in the lungs (**m**) and mammary glands (**o**), and immunohistochemistry in the lungs (**n**) and mammary glands (**p**) of control cows that were challenged with the DC/24 virus. H&E staining in the lungs (**q**) and mammary glands (**s**), and immunohistochemistry in the lungs (**r**) and mammary glands (**t**) of vaccinated cows that were challenged with the DC/24 virus. Original magnifications: ×400 (**a**, **c**, **e**, **g**, **i**, **k**, **m**, **o**, **q**, and **s**); ×100 (**b**, **d**, **f**, **h**, **j**, **l**, **n**, **p**, **r**, and **t**).

**Supplementary Figure S12. Protective efficacy of different vaccines in cattle against DC/24 virus challenge.** Vaccinated cattle and control cattle were challenged with DC/24 virus through both intranasal (2×10^6^ EID_50_) and intramammary gland (2×10^2^ EID_50_) inoculation, and tissues were collected for virus titration in eggs. Viral titers in tissues of cattle euthanized on days 3 (**a**), 6 (**b**), and 12 (**c**) post-challenge (p.c). Each color bar represents the value of an individual animal. The horizontal dashed lines in the panels indicate the lower limit of detection.

**Supplementary Figure S13. Receptor-binding preference analysis of H5N1 virus.** Two types of biotinylated glycans, Neu5Ac (α2-3)Gal (β1-4)GlcNAc (3′SLN) and Neu5Ac (α2-6)Gal (β1-4)GlcNAc (6′SLN), were used in this study. The binding interaction was monitored at 30°C for 500 seconds.

**Supplementary Table S1. Nucleotide and amino acid sequence identity among H5N1 viruses in this study.**

| Gene | Identity (%) | | | | | |
| --- | --- | --- | --- | --- | --- | --- |
|  | TS/23 vs DC/24 | | DK/24 vs DC/24 | | TS/23 vs DK/24 | |
|  | Nucleotide | Amino acid | Nucleotide | Amino acid | Nucleotide | Amino acid |
| HA | 97.5 | 98.9 | 96.4 | 98.2 | 97.7 | 98.9 |
| NA | 97.2 | 96.2 | 96.2 | 96 | 97.6 | 97.2 |
| PB2 | 83.7 | 97.8 | 84 | 97 | 90.2 | 97.4 |
| PB1 | 87.7 | 98.9 | 87.6 | 98.2 | 94.3 | 98.9 |
| PA | 97.7 | 97.9 | 95.1 | 98.2 | 95.4 | 98.0 |
| NP | 87.8 | 98.8 | 88.5 | 99 | 93.3 | 99.0 |
| M1 | 97.1 | 98 | 97.6 | 98 | 98.9 | 100 |
| M2 | 99.3 | 98 | 99 | 96.9 | 99.7 | 99.0 |
| NS1 | 92.5 | 95.2 | 92.6 | 95.2 | 94.5 | 94.8 |
| NS2 | 95.4 | 97.6 | 94.5 | 96.1 | 96.3 | 96.1 |

**Supplementary Table S2. Changes in body temperature and milk color in dairy cattle after infection with different viruses.**

| Infection route (dose) | Virus | Euthanized time [Day post infection (p.i.)] | Time of fever detected (Day p.i.) | Temperature increased (℃)^*^ | Milk color in mammary gland (Day p.i.)^&^ | | | |
| --- | --- | --- | --- | --- | --- | --- | --- | --- |
|  |  |  |  |  | Left front | Left rear | Right front | Right rear |
| Intranasal  (2×10^6^EID_50_) | TS/23 | 3 | / | / | Normal | Normal | Normal | Normal |
|  |  | 6 | / | / | Normal | Normal | Normal | Normal |
|  |  | 14 | / | / | Normal | Normal | Normal | Normal |
|  | DK/24 | 3 | / | / | Normal | Normal | Normal | Normal |
|  |  | 6 | / | / | Normal | Normal | Normal | Normal |
|  |  | 14 | / | / | Normal | Normal | Normal | Normal |
|  | DC/24 | 3 | / | / | Normal | Normal | Normal | Normal |
|  |  | 6 | 4 | 1.1 | Normal | Normal | Normal | Normal |
|  |  | 14 | / | / | Normal | Normal | Normal | Normal |
| Intramammary gland  (2×10^2^EID_50_) | TS/23 | 3 | / | / | Yellow (3) | Normal | Normal | Normal |
|  |  | 6 | 2 and 3 | 1.9; 0.8 | Yellow (4 to 6) | Normal | Normal | Yellow (3 to 6) |
|  |  | 14 | 2 | 1.2 | Yellow (4 to 6) | Normal | Normal | Yellow (4 to 6) |
|  | DC/24 | 3 | 2 and 3 | 0.9; 0.7 | Yellow (2 and 3) | Normal | Normal | Yellow (2 and 3) |
|  |  | 6 | 2 and 3 | 1.2; 0.8 | Yellow (2 to 6) | Normal | Normal | Yellow (4 to 6) |
|  |  | 14 | / | / | Yellow (3 to 7) | Normal | Normal | Yellow (3 to 7) |
| Intramammary gland** | DC/24 | 6 | 2, 3 and 4 | 2.0; 1.2; 0.5 | Normal | Normal | Yellow (5 to 6) | Yellow (3 to 6) |
|  |  | 6 | 2 | 2.0 | Normal | Normal | Normal | Yellow (3 to 6) |

*, Rectal temperatures were measured. The temperature increase was calculated by subtracting the average temperature over the three days prior to inoculation. /, Not detected.

^&^, Yellow milk was thicker than the normal milk.

**, Cattle were infected with three different doses of the virus to three different mammary glands: one EID_50_ in 1 ml to left rear, 10 EID_50_ in 1 ml to right front, and 10^2^EID_50_ in 1 ml to right rear.

**Supplementary Table S3. Body temperature change in calves and cows and milk color alteration of cows in the “mouth-to-teat” transmission study.**

| Room | Infection route | Cattle age | Time of fever detected  (Day p.i.) | Temperature  increased (℃)* | Milk color in mammary gland (Day p.i.)& | | | |
| --- | --- | --- | --- | --- | --- | --- | --- | --- |
|  |  |  |  |  | Left front | Left rear | Right front | Right rear |
| 1 | Not infected | 1. year-old   lactating cow | / | / | Normal | Normal | Normal | Normal |
|  | Intranasally | 4-week-old calf | 3 | 0.5 | N.A. | N.A. | N.A. | N.A. |
|  |  | 4-week-old calf | / | / | N.A. | N.A. | N.A. | N.A. |
| 2 | Not infected | 1. year-old   lactating cow | 10 | 2.1 | Normal | Normal | Normal | Yellow (10 to 12) |
|  | Orally | 5-week-old calf | 2 and 3 | 0.5; 0.5 | N.A. | N.A. | N.A. | N.A. |
|  |  | 4-week-old calf | 4 | 0.5 | N.A. | N.A. | N.A. | N.A. |

*, Rectal temperatures were measured. The temperature increase was calculated by subtracting the average temperature over the three days prior to inoculation. /, Not detected. N.A., not applicable.

^&^, Yellow milk was thicker than the normal milk.

**Supplementary Table S4. Antibody response in serum and milk of dairy cattle that received different vaccines.**

| Vaccine Group^*^ | Cattle age (year) | Hemagglutination inhibition (HI) and neutralization (NT) antibody titer in serum  at different weeks post-vaccination (HI/NT) | | | | | | | | Neutralization (NT) antibody titer in milk  at different weeks post-vaccination^&^ | | | | | | | | Challenge  virus |
| --- | --- | --- | --- | --- | --- | --- | --- | --- | --- | --- | --- | --- | --- | --- | --- | --- | --- | --- |
|  |  | 1 | 2 | 3 | 4 | 5 | 6 | 7 | 8 | 1 | 2 | 3 | 4 | 5 | 6 | 7 | 8 |  |
| Inactivated vaccine | 3 | <2/<2 | 32/64 | 32/64 | 128/256 | 256//512 | / | / | / | <2 | 4 | 8 | 32 | 64 | / | / | / | TS/23 |
|  | 3 | <2/<2 | 64/64 | 128/128 | 256/512 | 256/512 | / | / | / | <2 | 16 | 32 | 64 | 128 | / | / | / | TS/23 |
|  | 3 | <2/<2 | 32/64 | 128/256 | 128/256 | 512/512 | / | / | / | <2 | 8 | 16 | 32 | 64 | / | / | / | TS/23 |
|  | 4 | <2/<2 | 32/64 | 128/256 | 256/512 | 256/512 | 256/512 | 256/512 | 128/256 | <2 | 16 | 32 | 64 | 64 | 64 | 64 | 64 | DC/24 |
|  | 3 | <2/<2 | 32/64 | 64/128 | 256/512 | 512/512 | 512/1024 | 256/512 | 256/512 | <2 | 4 | 16 | 64 | 128 | 128 | 128 | 64 | DC/24 |
|  | 3 | <2/<2 | 32/64 | 64/128 | 256/512 | 256/512 | 256/512 | 256/512 | 256/512 | <2 | 8 | 16 | 32 | 128 | 128 | 128 | 64 | DC/24 |
|  | 3 | <2/<2 | 16/32 | 64/128 | 128/128 | 256/512 | / | / | / | <2 | 2 | 8 | 16 | 32 | / | / |  | DC/24 |
|  | 3 | <2/<2 | 32/32 | 64/128 | 256/512 | 256/512 | / | / | / | <2 | 4 | 16 | 32 | 64 | / | / | / | DC/24 |
|  | 3 | <2/<2 | 32/64 | 128/256 | 256/512 | 256/512 | / | / | / | <2 | 2 | 16 | 32 | 64 | / | / | / | DC/24 |
| DNA vaccine | 6 | <2/<2 | 4/8 | 8/16 | 64/128 | 64/128 | / | / | / | <2 | 4 | 4 | 4 | 32 | / | / | / | TS/23 |
|  | 3 | <2/<2 | <2/<2 | 8/16 | 64/128 | 64/128 | / | / | / | <2 | <2 | 8 | 8 | 64 | / | / | / | TS/23 |
|  | 3 | <2/<2 | 4/8 | 8/8 | 64/128 | 128/256 | / | / | / | <2 | <2 | 4 | 8 | 32 | / | / | / | TS/23 |
|  | 7 | <2/<2 | 4/8 | 4/8 | 32/128 | 32/64 | 64/128 | 64/128 | 64/128 | <2 | <2 | 4 | 8 | 32 | 32 | 32 | 64 | DC/24 |
|  | 4 | <2/<2 | <2/<2 | 4/8 | 64/128 | 64/64 | 64/128 | 32/64 | 32/64 | <2 | <2 | <2 | 4 | 32 | 64 | 64 | 32 | DC/24 |
|  | 4 | <2/<2 | <2/<2 | 4/8 | 64/128 | 64/128 | 64/128 | 64/128 | 32/64 | <2 | <2 | 4 | 8 | 32 | 32 | 32 | 32 | DC/24 |
|  | 3 | <2/<2 | <2/2 | 4/8 | 32/64 | 64/128 | / | / | / | <2 | <2 | <2 | 4 | 32 | / | / | / | DC/24 |
|  | 3 | <2/<2 | 2/2 | 4/8 | 64/128 | 64/128 | / | / | / | <2 | <2 | 2 | 4 | 32 | / | / | / | DC/24 |
|  | 3 | <2/<2 | 2/4 | 8/32 | 64/128 | 128/256 | / | / | / | <2 | <2 | 2 | 4 | 64 | / | / | / | DC/24 |
| Control | 3 | <2/<2 | <2/<2 | <2/<2 | <2/<2 | <2/<2 | / | / | / | <2 | <2 | <2 | <2 | <2 | / | / | / | TS/23 |
|  | 3 | <2/<2 | <2/<2 | <2/<2 | <2/<2 | <2/<2 | / | / | / | <2 | <2 | <2 | <2 | <2 | / | / | / | TS/23 |
|  | 3 | <2/<2 | <2/<2 | <2/<2 | <2/<2 | <2/<2 | / | / | / | <2 | <2 | <2 | <2 | <2 | / | / | / | TS/23 |
|  | 3 | <2/<2 | <2/<2 | <2/<2 | <2/<2 | <2/<2 | <2/<2 | <2/<2 | <2/<2 | <2 | <2 | <2 | <2 | <2 | <2 | <2 | <2 | DC/24 |
|  | 3 | <2/<2 | <2/<2 | <2/<2 | <2/<2 | <2/<2 | <2/<2 | <2/<2 | <2/<2 | <2 | <2 | <2 | <2 | <2 | <2 | <2 | <2 | DC/24 |
|  | 3 | <2/<2 | <2/<2 | <2/<2 | <2/<2 | <2/<2 | <2/<2 | <2/<2 | <2/<2 | <2 | <2 | <2 | <2 | <2 | <2 | <2 | <2 | DC/24 |
|  | 3 | <2/<2 | <2/<2 | <2/<2 | <2/<2 | <2/<2 | / | / | / | <2 | <2 | <2 | <2 | <2 | / | / | / | DC/24 |
|  | 4 | <2/<2 | <2/<2 | <2/<2 | <2/<2 | <2/<2 | / | / | / | <2 | <2 | <2 | <2 | <2 | / | / | / | DC/24 |
|  | 4 | <2/<2 | <2/<2 | <2/<2 | <2/<2 | <2/<2 | / | / | / | <2 | <2 | <2 | <2 | <2 | / | / | / | DC/24 |

*, Cattle were inoculated with two doses of the indicated vaccine, at a 3-week interval. Antibody titers were measured by using the vaccine strain H5-Re14.

^&^, Due to non-specific inhibitory interference, HI antibodies in milk were not determined.

/, Not available.

**Supplementary Table S5. Antibody titers in cattle induced by different virus infection.**

| Virus | Inoculation route (dose) | Test time [(day post-infection) (p.i.)]* | HI antibody titer in serum | NT antibody titer in serum | NT antibody titer in milk from different utter** | | | |
| --- | --- | --- | --- | --- | --- | --- | --- | --- |
|  |  |  |  |  | Left front | Left rear | Right front | Right rear |
| TS/23 | Intranasal inoculation (2 × 10^6^ EID_50_) | 14 p.i. | 16 | 32 | 4 | 4 | 4 | 4 |
| DK/24 | Intranasal inoculation (2 × 10^6^ EID_50_) | 14 p.i. | 8 | 16 | <2 | <2 | <2 | <2 |
| DC/24 | Intranasal inoculation (2 × 10^6^ EID_50_) | 14 p.i. | 16 | 64 | 8 | 8 | 8 | 8 |
| TS/23 | Intramammary gland inoculation (2 × 10^2^ EID_50_) | 14 p.i. | 8 | 16 | 8 | <2 | <2 | 8 |
| DC/24 | Intramammary gland inoculation (2 × 10^2^ EID_50_) | 14 p.i. | 16 | 32 | 8 | <2 | <2 | 4 |
| TS/23 | Intranasal (2 × 10^6^ EID_50_) and  intramammary gland challenge (2 × 10^2^ EID_50_) | 12 p.i. | 8 | 16 | 4 | <2 | <2 | 4 |
| DC/24 | Intranasal (2 × 10^6^ EID_50_) and  intramammary gland challenge (2 × 10^2^ EID_50_) | 12 p.i. | 32 | 64 | 8 | <2 | <2 | 8 |
| DC/24 | Intramammary gland challenge (10^2^ ,10^4^ and 10^6^ EID_50_) | 12 p.i. | 32 | 64 | <2 | 4 | 8 | 8 |

*, Animals tested on day 14 p.i. were the ones used for the infection studies, and animals tested on day 12 p.i. were the control animals in the vaccine protective studies. Antibody titers were measured by using the homologous virus.

**, Due to non-specific inhibitory interference, HI antibodies in milk were not determined.

**Supplementary Table S6. Hemagglutination inhibition (HI) and neutralization (NT) antibody titers of dairy cattle induced by different vaccines against the challenge virus*.**

| Challenge  virus | Challenge route | Vaccine | Cattle age (year) | Time for sample collection  (Weeks  post-2nd dose) | HI antibody  titer in serum | NT antibody  titer in serum | NT antibody titer  in milk |
| --- | --- | --- | --- | --- | --- | --- | --- |
| TS/23 | Intranasal and intramammary gland | Inactivated | 3 | 2 | 256 | 512 | 64 |
|  |  |  | 3 | 2 | 256 | 512 | 128 |
|  |  |  | 3 | 2 | 256 | 512 | 128 |
|  |  | DNA | 6 | 2 | 64 | 128 | 32 |
|  |  |  | 3 | 2 | 64 | 128 | 32 |
|  |  |  | 3 | 2 | 64 | 256 | 32 |
| DC/24 | Intranasal and intramammary gland | Inactivated | 4 | 5 | 256 | 512 | 32 |
|  |  |  | 3 | 5 | 256 | 512 | 64 |
|  |  |  | 3 | 5 | 128 | 256 | 64 |
|  |  | DNA | 7 | 5 | 64 | 128 | 32 |
|  |  |  | 4 | 5 | 32 | 128 | 32 |
|  |  |  | 4 | 5 | 32 | 128 | 32 |
|  | Intramammary gland | Inactivated | 3 | 2 | 128 | 256 | 32 |
|  |  |  | 3 | 2 | 128 | 512 | 32 |
|  |  |  | 3 | 2 | 128 | 256 | 64 |
|  |  | DNA | 3 | 2 | 64 | 128 | 32 |
|  |  |  | 3 | 2 | 64 | 128 | 64 |
|  |  |  | 3 | 2 | 64 | 256 | 64 |

*, Data shown are from serum and milk samples collected before challenge. Due to non-specific inhibitory interference, HI antibodies in milk were not determined.

**Supplementary Table S7. Changes in body temperature and milk of cattle post-challenge (p.c.).**

| Challenge virus | Challenge route* | Group | Euthanized time  (Day p.c.) | Time of fever detected  (Day p.c.) | Temperature increased (℃)** | Color of milk collected from different mammary glands (Day p.c.)& | | | |
| --- | --- | --- | --- | --- | --- | --- | --- | --- | --- |
|  |  |  |  |  |  | Left front | Left rear | Right front | Right rear |
| TS/23 | Intranasal and intramammary gland | Control | 3 | 2 | 2.3 | Yellow (3) | Normal | Normal | Yellow (3) |
|  |  |  | 6 | 3 | 2 | Yellow (3 to 6) | Normal | Normal | Yellow (3 to 6) |
|  |  |  | 12 | / | / | Yellow (3 to 6) | Normal | Normal | Yellow (3 to 7) |
|  |  | Inactivated vaccine | 3 | / | / | Normal | Normal | Normal | Normal |
|  |  |  | 6 | / | / | Normal | Normal | Normal | Normal |
|  |  |  | 12 | / | / | Normal | Normal | Normal | Normal |
|  |  | DNA vaccine | 3 | / | / | Normal | Normal | Normal | Normal |
|  |  |  | 6 | / | / | Normal | Normal | Normal | Normal |
|  |  |  | 12 | / | / | Normal | Normal | Normal | Normal |
| DC/24 | Intranasal and intramammary gland | Control | 3 | 2 and 3 | 0.7; 0.7 | Normal | Normal | Normal | Yellow (3) |
|  |  |  | 6 | / | / | Yellow (3 to 6) | Normal | Normal | Yellow (3 to 6) |
|  |  |  | 12 | 3 | 2 | Yellow (4 to 7) | Normal | Normal | Yellow (5 to 7) |
|  |  | Inactivated vaccine | 3 | / | / | Normal | Normal | Normal | Normal |
|  |  |  | 6 | / | / | Normal | Normal | Normal | Normal |
|  |  |  | 12 | / | / | Normal | Normal | Normal | Normal |
|  |  | DNA vaccine | 3 | / | / | Normal | Normal | Normal | Normal |
|  |  |  | 6 | / | / | Normal | Normal | Normal | Normal |
|  |  |  | 12 | / | / | Normal | Normal | Normal | Normal |
| DC/24 | Intra-  mammary gland | Control | 3 | 1 | 1.2 | Normal | Normal | Yellow (3) | Yellow (2 and 3) |
|  |  |  | 6 | 1, 2 and 3 | 2.3; 1.0; 0.9 | Normal | Yellow (4 to 6) | Yellow (3 to 6) | Yellow (2 to 6) |
|  |  |  | 12 | / | / | Normal | Yellow (4 to 8) | Yellow (4 to 7) | Yellow (2 to 8) |
|  |  | Inactivated vaccine | 3 | / | / | Normal | Normal | Normal | Normal |
|  |  |  | 6 | / | / | Normal | Normal | Normal | Normal |
|  |  |  | 12 | / | / | Normal | Normal | Normal | Normal |
|  |  | DNA vaccine | 3 | / | / | Normal | Normal | Normal | Normal |
|  |  |  | 6 | / | / | Normal | Normal | Normal | Normal |
|  |  |  | 12 | / | / | Normal | Normal | Normal | Normal |

*, Intranasal and intramammary gland challenge: 2 × 10^6^ EID_50_ of the virus was administered intranasally (1 × 10^6^ EID_50_ per nostril) and 2 × 10^2^ EID_50_ of the virus was administered into the left front and right rear mammary glands (1 × 10^2^ EID_50_ per gland); Intramammary gland challenge: the left rear, right front, and right rear mammary glands of cattle were, respectively, inoculated with 10^2^, 10^4^, and 10^6^ EID_50_ of the virus.

**, Rectal temperatures were measured. The temperature increase was calculated by subtracting the average temperature over the three days prior to challenge. /, Not detected.

^&^, Color change was not observed in the milk collected from the uninfected mammary glands. Yellow milk was thicker than the milk produced by uninfected mammary glands.

**Supplementary Table S8. Amino acid substitution identified by next-generation sequencing in samples collected from cattle infected with the TS/23 virus.**

| Virus | Protein | Amino acid at the indicated position | Substituent composition (%)^#^ | |
| --- | --- | --- | --- | --- |
|  |  |  | Nasal swab | Milk |
| TS/23 | PB2 | E358 | G (5.52%); A (2.40%); V (1.55%) | G (4.81%); V (1.20%); A (1.09%) |
|  |  | R641 | G (3.17%); W (1.84%) | G (3.58%); W (1.82%) |
|  | PA | W368 | R (5.36%); G (2.15%) | R (6.28%); G (2.09%) |
|  |  | M595 | I (99.82%) | I (95.66%) |
|  | HA | I335 (H3 numbering) | M (21.28%) | M (4.25%) |
|  | NP | W386 | K (4.89%); G (3.22%) | R (3.37%); G (1.83%) |
|  | NA | E77 | G (6.5%) | G (1.47%) |
|  |  | S95 | R (7.67%); G (3.30%) | R (2.07%) |
|  | M1 | K57 | R (6.43%); T (4.62%); M (3.49%) | R (3.85%); T (2.08%); M (1.74%) |

^#^, Only positions with substitutions accounting for > 5% are shown. For positions with different substitutions, only those with individual frequencies >1% are displayed. Nasal swabs were collected from cattle on day 6 p.i. and milk samples were collected from cattle on day 9 p.i.
