# Supplementary figures and images for: H5N1 virus invades the mammary glands of dairy cattle through ‘mouth-to-teat’ transmission
Source: Natl Sci Rev. 2025 Jul 1;12(9):nwaf262. doi: 10.1093/nsr/nwaf262 (PMC12342610; doi:10.1093/nsr/nwaf262)

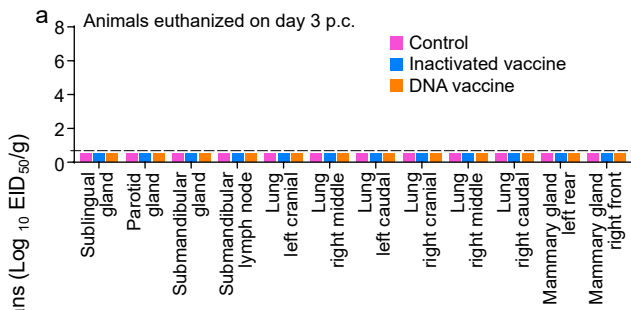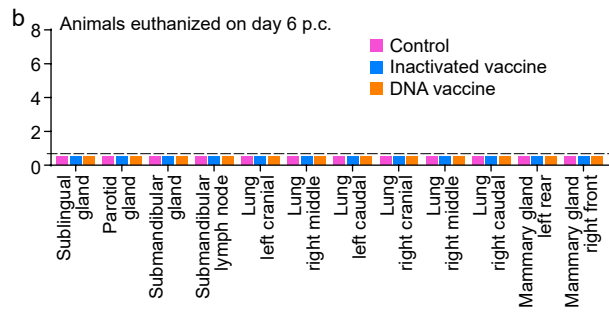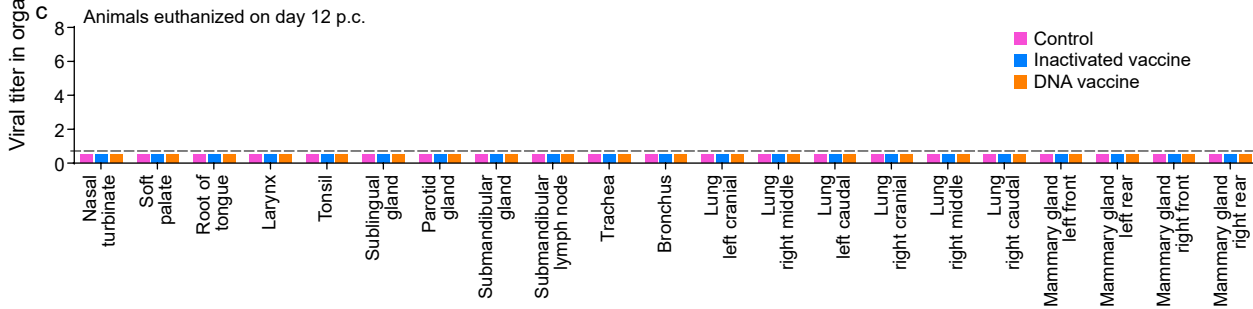

Supplement: nwaf262_Supplemental_Files [file nwaf262_supplemental_files.zip › Shi_Fig_S10.pdf]

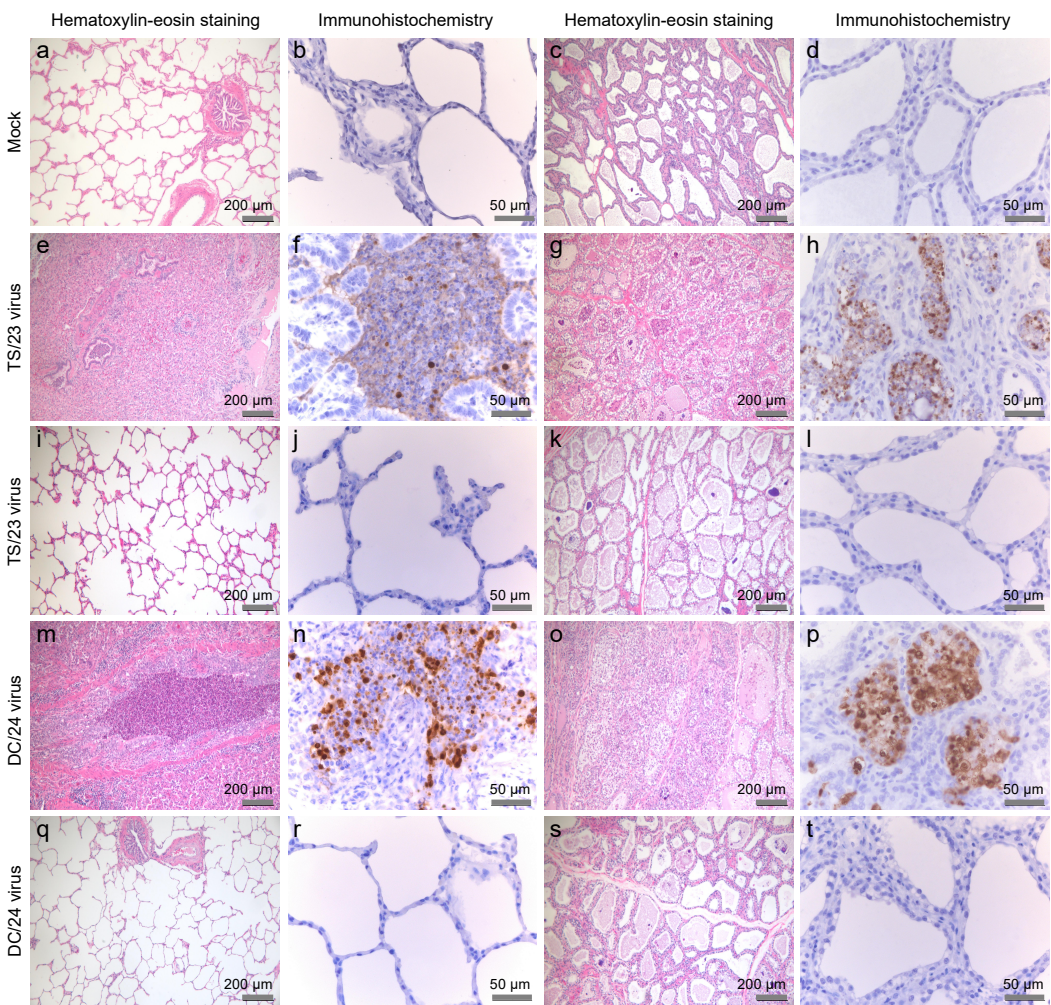

Supplement: nwaf262_Supplemental_Files [file nwaf262_supplemental_files.zip › Shi_Fig_S11.pdf]

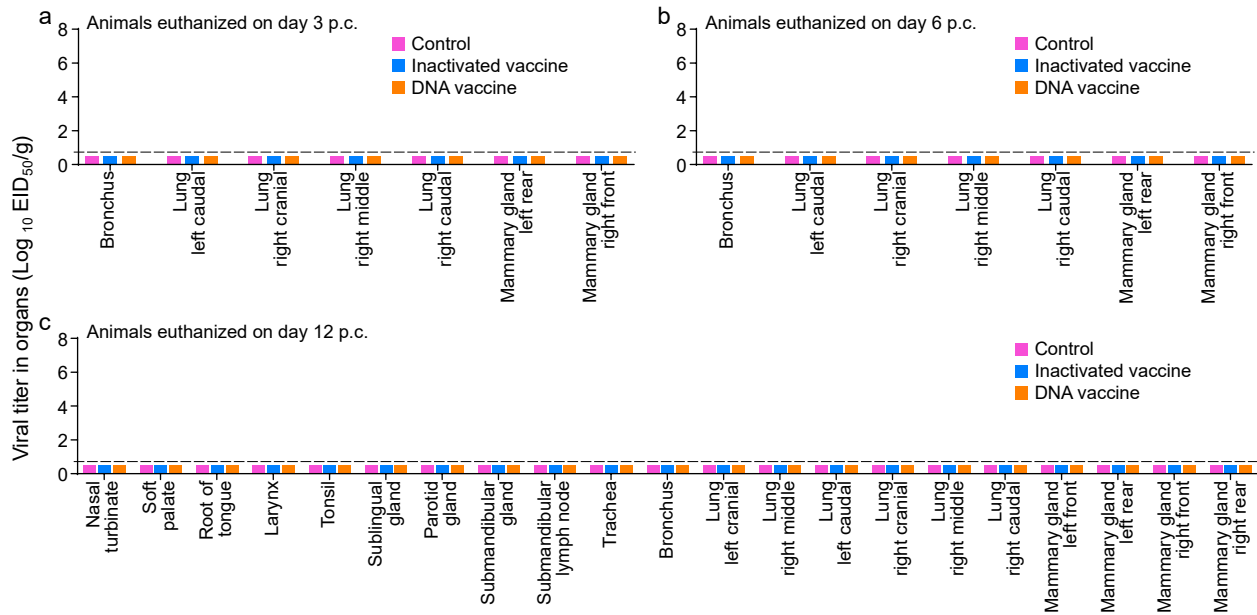

Supplement: nwaf262_Supplemental_Files [file nwaf262_supplemental_files.zip › Shi_Fig_S12.pdf]

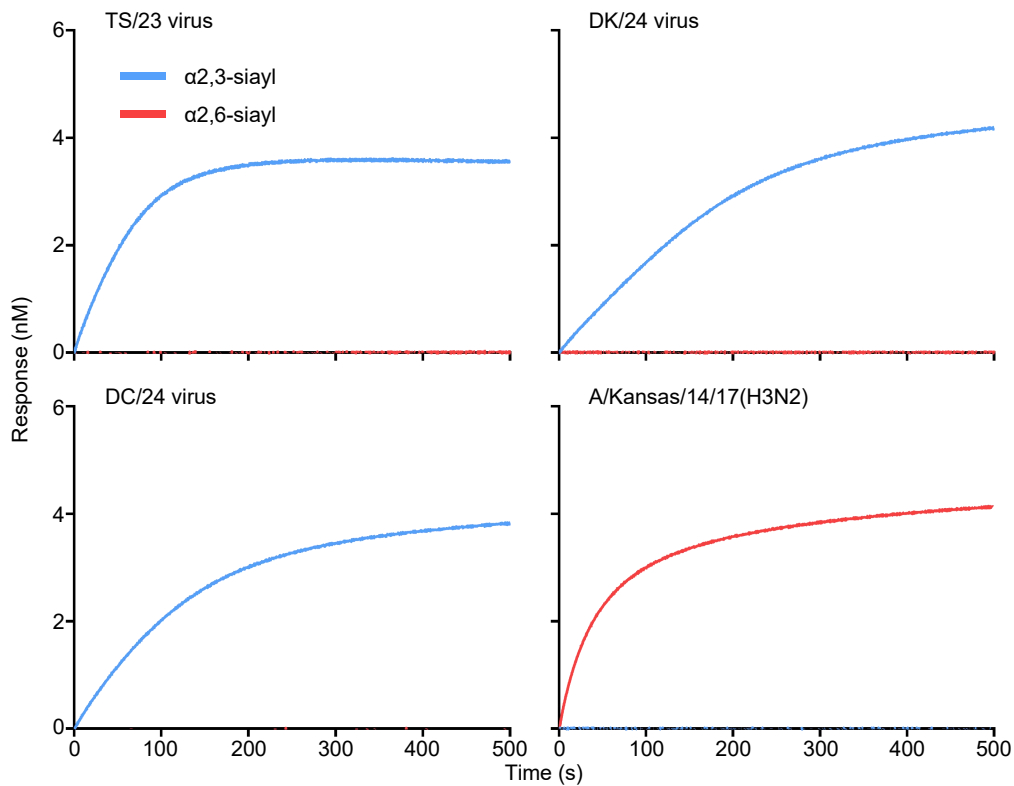

Supplement: nwaf262_Supplemental_Files [file nwaf262_supplemental_files.zip › Shi_Fig_S13.pdf]

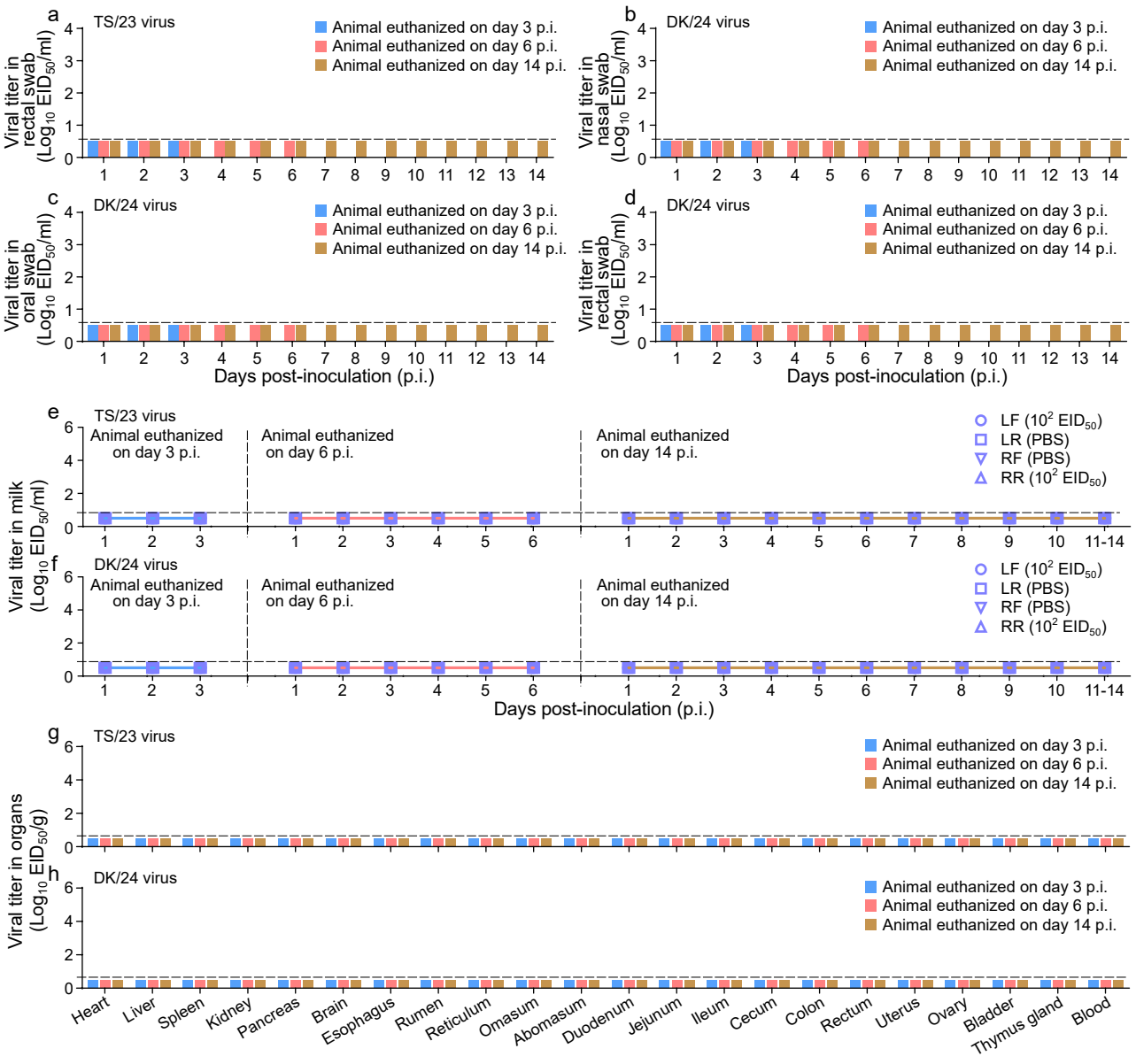

Supplement: nwaf262_Supplemental_Files [file nwaf262_supplemental_files.zip › Shi_Fig_S2.pdf]

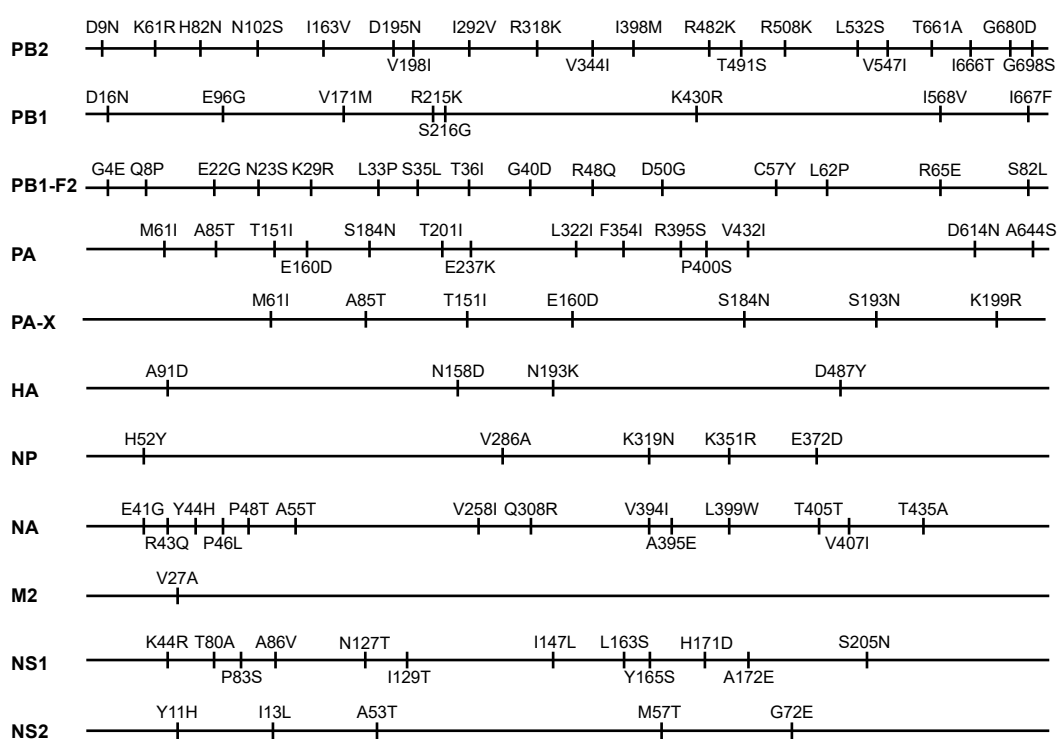

Supplement: nwaf262_Supplemental_Files [file nwaf262_supplemental_files.zip › Shi_Fig_S3.pdf]

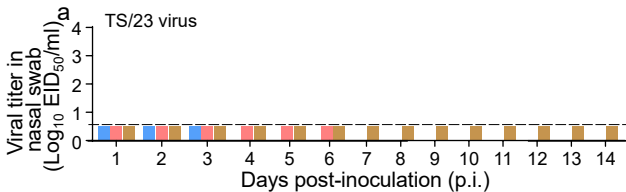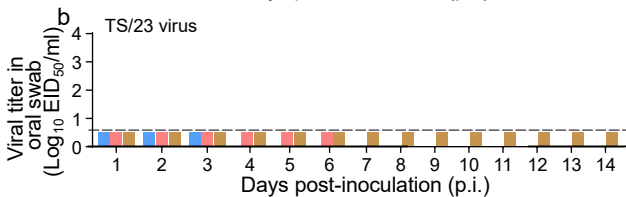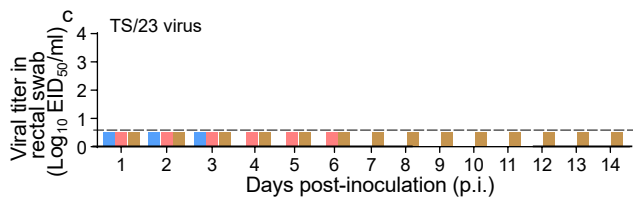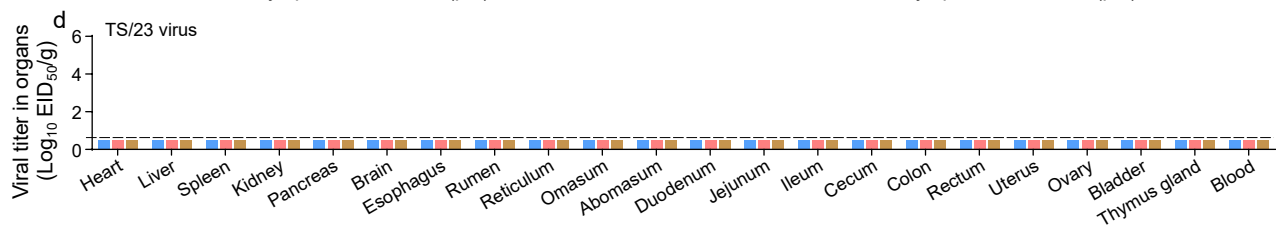

- Animal euthanized on day 3 p.i.
- Animal euthanized on day 6 p.i.
- Animal euthanized on day 14 p.i.

Supplement: nwaf262_Supplemental_Files [file nwaf262_supplemental_files.zip › Shi_Fig_S4.pdf]

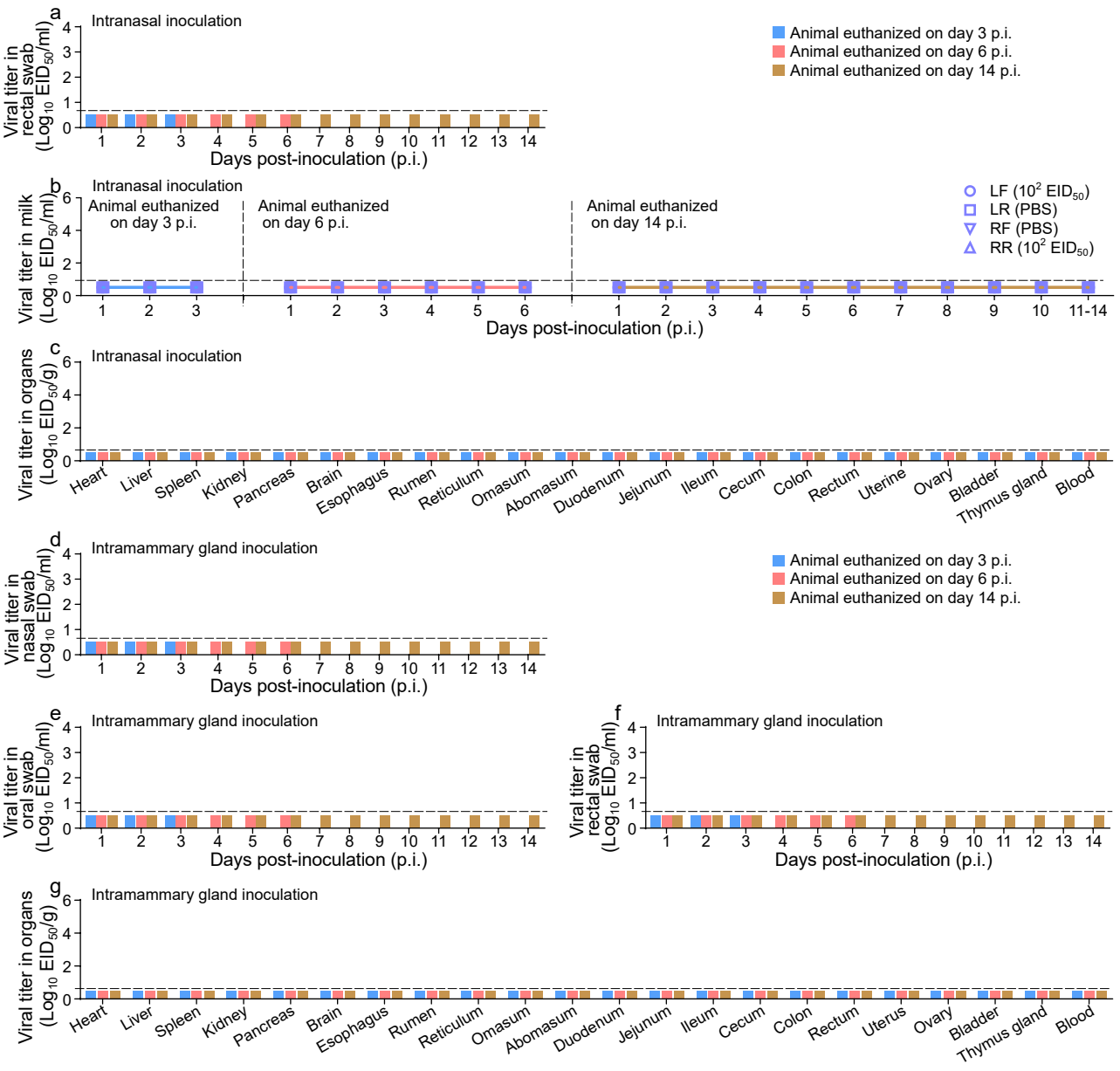

Supplement: nwaf262_Supplemental_Files [file nwaf262_supplemental_files.zip › Shi_Fig_S5.pdf]

**MAL-I**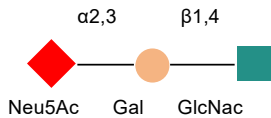**MAL-II**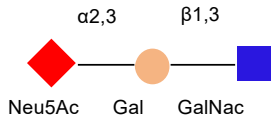**SNA**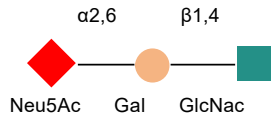**Trachea**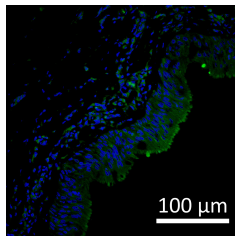**Lung**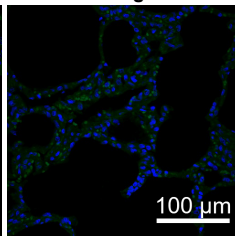**Mammary gland**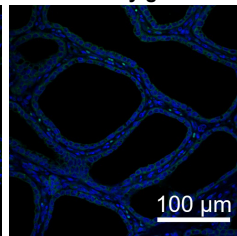**MAL-I**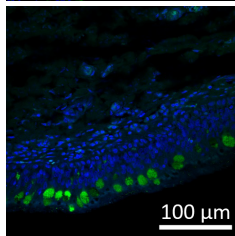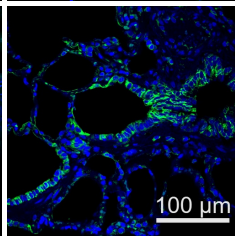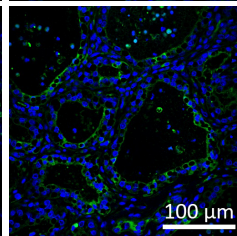**MAL-II**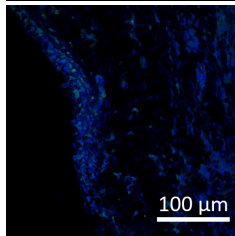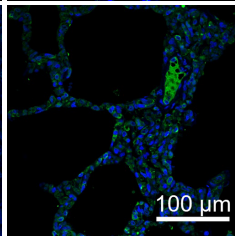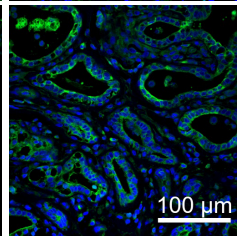**SNA**

Supplement: nwaf262_Supplemental_Files [file nwaf262_supplemental_files.zip › Shi_Fig_S6.pdf]

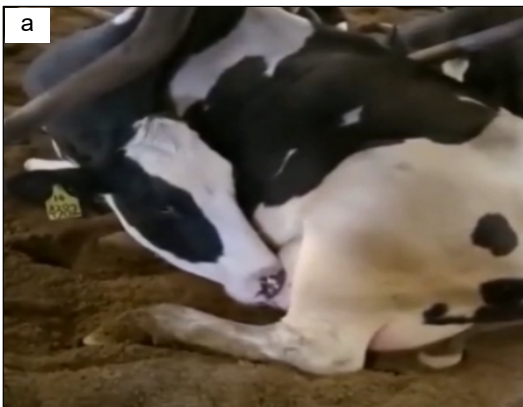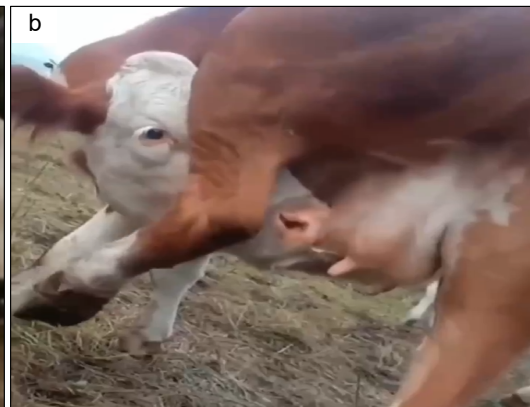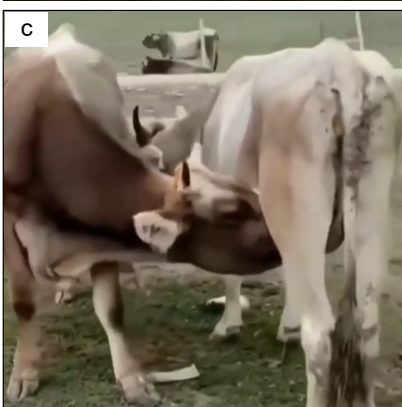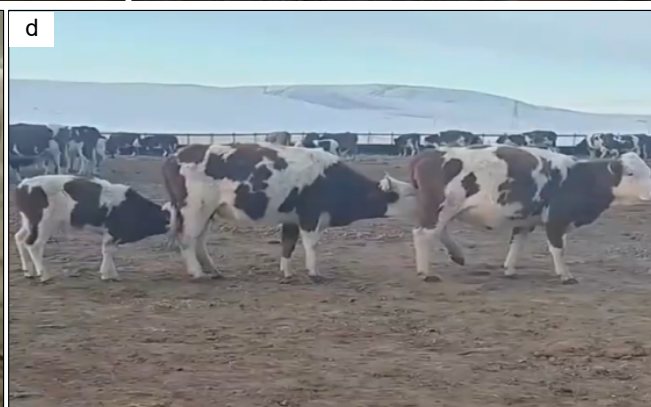

Supplement: nwaf262_Supplemental_Files [file nwaf262_supplemental_files.zip › Shi_Fig_S7.pdf]

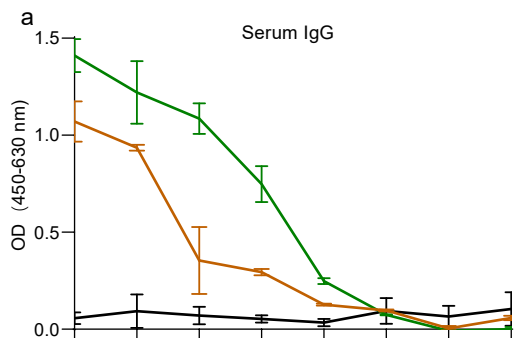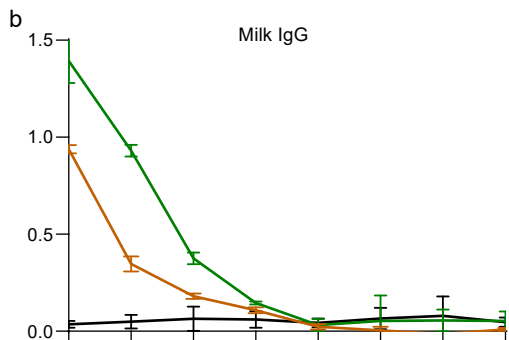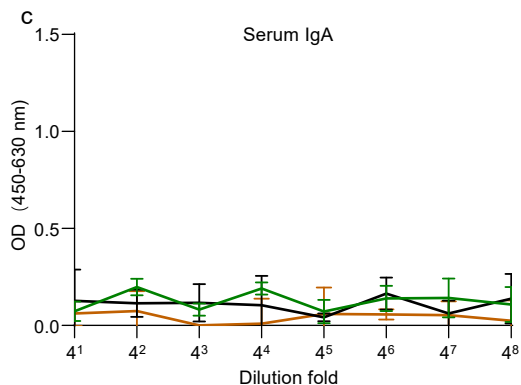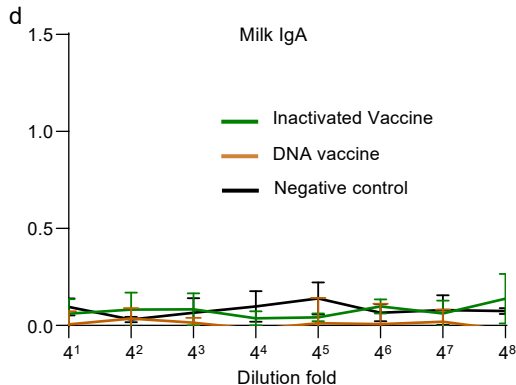

Supplement: nwaf262_Supplemental_Files [file nwaf262_supplemental_files.zip › Shi_Fig_S9.pdf]
